# Breastfeeding practices, beliefs, and social norms in low-resource communities in Mexico: Insights for how to improve future promotion strategies

**DOI:** 10.1371/journal.pone.0180185

**Published:** 2017-07-03

**Authors:** Tessa M. Swigart, Anabelle Bonvecchio, Florence L. Théodore, Sophia Zamudio-Haas, Maria Angeles Villanueva-Borbolla, James F. Thrasher

**Affiliations:** 1 Nutrition and Health Research Center, National Institute of Public Health. Cuervanaca, Morelos, Mexico; 2 Center for AIDS Prevention Studies, University of California, San Francisco. California, United States of America; 3 Department of Health Promotion, Education, and Behavior, University of South Carolina, Columbia, South Carolina, United States of America; TNO, NETHERLANDS

## Abstract

**Introduction:**

Breastfeeding is recommended exclusively for the first 6 months after birth, with continued breastfeeding for at least 2 years. Yet prevalence of these recommendations is low globally, although it is an effective and cost-effective way to prevent serious infections and chronic illness. Previous studies have reported that social support greatly influences breastfeeding, but there is little evidence on perceived social norms in Mexico and how they affect actual behavior.

**Objective:**

Our objective was to investigate breastfeeding intention, practices, attitudes, and beliefs, particularly normative, among low-resource communities in central and southern Mexico.

**Methods:**

We performed a secondary analysis using the theory of planned behavior with cross-sectional data, which included semi-structured individual interviews with fathers (*n* 10), 8 focus groups with mothers (*n* 50), and 8 focus groups with women community leaders (*n* 44) with a total of 104 participants. Our data also included a quantitative survey among pregnant women and mothers (*n* 321).

**Results:**

Women reported supplementing breast milk with water and teas soon after birth, as well as introducing small bites of solid food a few months after birth. Social norms appeared to support breastfeeding, but not exclusive breastfeeding or breastfeeding for periods longer than about a year. This may be partially explained by: a) behavioral beliefs that for the first 6 months breast milk alone is insufficient for the baby, and that water in addition to breast milk is necessary to hydrate an infant and b) normative beliefs related to the appropriateness of breastfeeding in public and as the child gets older.

**Conclusions:**

Future strategies should focus on positively influencing social norms to support recommended practices, and emphasize the specific reasons behind the recommendations. Future efforts should take a multi-pronged approach using a variety of influences, not only directed at healthcare providers but close family members, including fathers.

## Introduction

The World Health Assembly and World Health Organization recommend exclusive breastfeeding (EBF) for the first 6 months of an infant’s life, with continued breastfeeding (BF) and complementary feeding introduced after the first 6 months up to age 2 years or more [1]. Compared to infants who are partially breastfed or not breastfed, infants with EBF for the first 6 months (i.e., without introduction of any other foods or liquids) experience better nutritional intake, fewer micronutrient deficiencies, fewer infectious episodes, fewer hospital visits, greater cognitive ability and lower mortality [2–5]. Additional evidence suggests a protective effect of BF in the prevention of childhood obesity [6] and mounting data indicate chronic disease protection lasting well-into adulthood, possibly reducing the likelihood of obesity, diabetes, and hypertension [7].

Recommended BF practices are a crucial and cost-effective way to help solve the dual burden of the nutritional transition in many low and middle-income countries (LMICs). The dual burden is characterized by malnutrition and micronutrient deficiencies in conjunction with chronic nutrition-related issues such as diabetes and obesity [8,9], and it most greatly impacts low-resource populations [10,11]. EBF for the first 6 months in LMICs globally is only 37%, and in Latin America the prevalence is lower than in other resource-poor areas [2]. Mexico has one of the lowest prevalence of EBF among all Latin American countries and it has dropped significantly; in 1999, EBF nationally was at 20.3%, but fell to 14.4% in 2012 [12]. The prevalence of EBF less than 6 months in rural and indigenous areas, where poverty is highest, has also shown the most dramatic decrease, dropping from 36.9% in 1999 to 18.5% in 2012 [13].

The Theory of Planned Behavior (TPB) is one of the most influential behavioral theories and has been used in previous studies to predict BF behavior [14–17]. The theory states that intention to perform a behavior is most proximal to engagement of behavior, and is based on an individual’s attitudes, social normative beliefs, and perceived behavioral control [18]. Fishbein’s updated version to TPB, the integrated model (IM) considers relevant skills and abilities, external barriers and facilitators, and an individual’s social and informational background [19]. Critics of the TPB have pointed out that intention alone is not sufficiently predictive when carrying out a behavior that relies greatly on both complex skills and also on strong social support, as for BF [20,21]. For example, Göksen found that intention was not a reliable determinant of BF except in the presence of both positive BF social support and normative beliefs [21]. Evidence from different LMICs has underscored the importance of a women’s social support within her family, community, and health care providers as well as institutional constituents such as policy and law [14,21–23].

Yet there is a need, especially given the importance of BF, the globally low rates, and dramatically decreased rates in Mexico, for greater understanding of various influences on BF intentions and practices in order to positively and effectively influence behavior. More specifically, there is insufficient research on BF normative beliefs; that is, what individuals perceive as appropriate BF behavior within their social networks, and how this affects actual practices. This study, through a secondary analysis, used the IM of the TPB as a framework to investigate BF intention, practices, attitudes, and beliefs, but in particular social norms, among low-resource communities in central and southern Mexico in order to understand how to better target and influence norms in future BF interventions.

## Materials and methods

### Original study design

**Fig 1:**
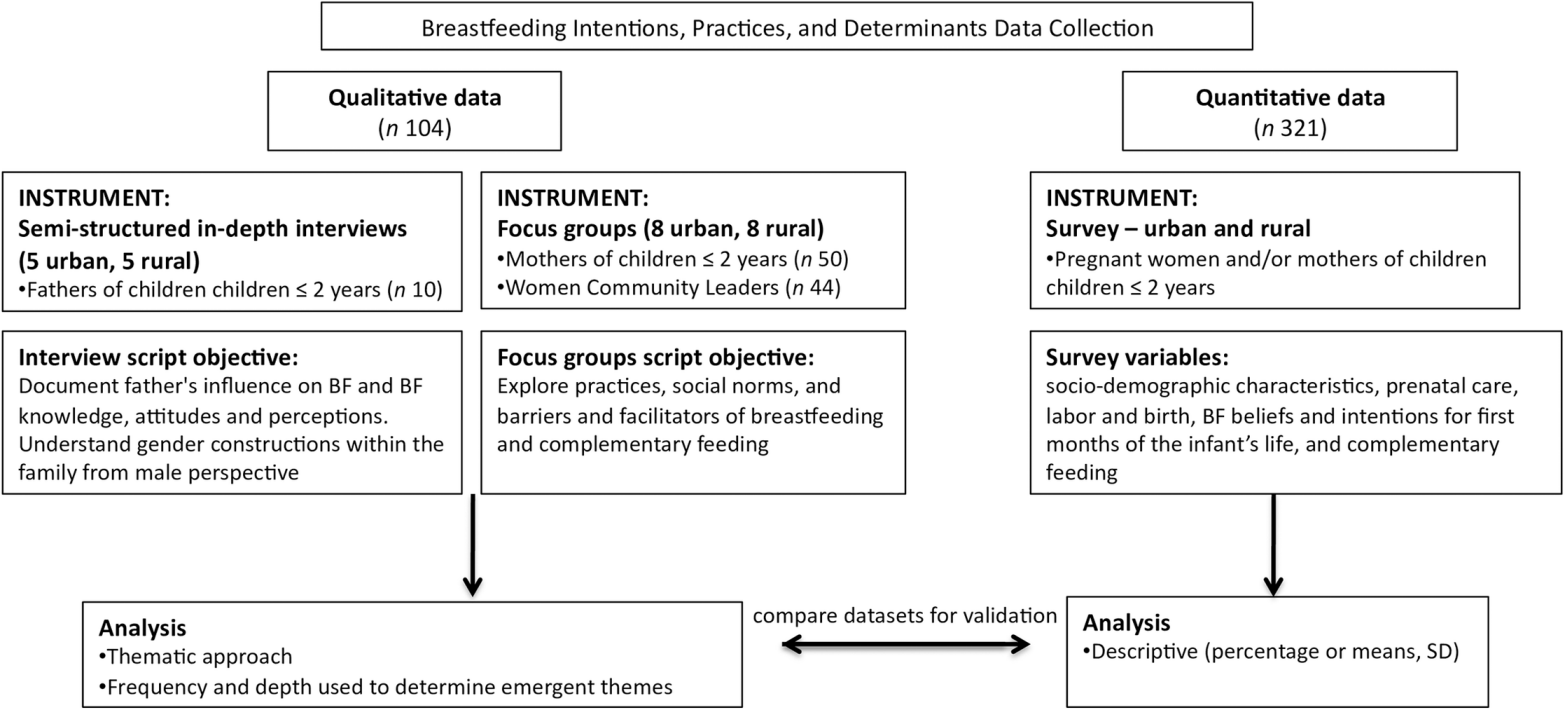
Flowchart of data collection, analysis, and dataset validation.

In 2013, a multidisciplinary team of researchers from the National Institute of Public Health in Mexico (INSP) conducted cross-sectional formative research in order to develop a social and behavior change interpersonal communication strategy to improve infant feeding practices among beneficiaries of Mexico’s poverty alleviation program, *Prospera*. *Prospera* (formerly *Oportunidades and Progresa*) was first developed in 1997 as a conditional cash transfer program for low-resource families, especially rural and indigenous populations, to create incentives for consistent school attendance, health clinic visits and improved nutrition [24]. For this paper, the authors conducted a secondary analysis using the qualitative and quantitative datasets from the formative research (Fig 1), and we used the consolidated criteria for reporting qualitative research (COREQ) checklist [25]. The formative research was from two states, Oaxaca and Queretaro, chosen to be as representative as possible of the populations who benefit from *Prospera*. Oaxaca is in the south, has the largest number of *Prospera* participants, and is one of the poorest states in Mexico. Queretaro is located in the center of the country and shares socio-demographic characteristics with other central states. Within both states, urban and rural areas were selected to improve representation. The data were collected from October through December 2013, and all participants consented to participate based on informed consent that was read orally as well as given in writing. The Ethics Committee at the National Institute of Public Health of Mexico approved this work (Approved September 18, 2013, CI: 1175, Folio Identification: F-73).

### Location and participant selection

For the quantitative data, ten locations were chosen in each state and were divided equally between urban (>15,000 inhabitants) or rural (<15,000 inhabitants). The urban and rural locations were purposively chosen based on the number of *Prospera* beneficiaries in the communities, and study personnel recruited participants by approaching them at local health clinics. Inclusion was limited to women from these communities who were pregnant or had children 2 years or younger. The survey instrument was designed based on the conceptual framework of the study by researchers from the INSP with previous experience on studies that shared similar objectives. The survey only included close-ended questions, and most sections of the survey were taken from previously validated surveys with pre-established responses [26–28]. Some of the questions in the segments of the survey pertaining to BF practices and social norms were developed from extensive qualitative formative research that the investigators carried out in 2008 among the study population. The survey instrument was pilot tested and further refined with a small subset of the target population. As is common in formative research that explores new social phenomena–and thus where previously validated data collection tools may not be available–we used triangulation of mixed methods in order to cross-validate our survey questions that had not been previously validated or verified for reproducibility (the survey is included as Appendix 1, although we did not use all sections for our secondary analysis) [29]. The survey was administered by experienced field workers who had been trained on the study’s methodology, who sat with each survey participant and recorded the answers after asking each question.

For the qualitative data, there were 2 locations, rural and urban, chosen for each state. The rural locations were between 60 and 80 minutes from each state’s capital, and the urban locations were located within each capital. These locations were purposively chosen based on number of beneficiaries and logistical convenience. In both states, mothers of children under two years were invited to participate from a list of the *Prospera* beneficiaries. Using snowball sampling and lists of *Prospera* beneficiaries, recruitment of women and fathers of children less than 2 years old was done with beneficiary support, managers of the local health centers, and women community leaders (mothers, mother-in-laws, and midwives) from each location. The community leaders were identified by personnel and participants in the local health centers as being important female figures in the community and were most commonly grandmothers or *promotoras* (community health workers). In some cases, because many of the fathers were away working during the day, some participants were recruited by going door to door in the community. Trained, male anthropologists carried out 10 in-depth interviews with the fathers (4 from rural Queretaro, 2 from urban Queretaro, 2 from rural Oaxaca, and 2 from urban Oaxaca). The researchers in charge of the design, field work, and primary data analysis for the qualitative data were from the INSP. The INSP was an independent institution contracted by *Prospera* to conduct the formative research, which helped to maintain objectivity and avoid bias. Two female doctorate-level trained researchers (one sociologist and one psychologist) designed the interview and focus group guides and each carried out 4 focus groups with mothers and 4 with community leaders in the state of Oaxaca (2 urban and 2 rural) and state of Quéretaro (2 urban and 2 rural). There were separate scripts for the interviews and focus groups, with differences in script between mothers and community leaders.They pre-tested each guide first in order to focus and refine them. The total number of interviews and focus groups were pre-established based on saturation from previous formative research conducted in 2008. The moderators took field notes during each focus group and communicated ideas and experiences to each other and the study teams throughout the process. Focus groups and interviews were performed in community centers or health clinics and were tape-recorded and transcribed verbatim. Each interview and focus group took 1.5–2 hours and participants were compensated with a small token gift (such as a baby bib or baby’s small bowl and spoon). All participants that were approached about participating in the data collection agreed. This could be due to the fact that *Prospera* beneficiaries are often asked to participate in program-sponsored activities and are used to offering personal information. The researchers assessed the potential for bias due to these conditions and concluded that they were minimal due to the study’s themes.

### Conceptual framework

The theory of planned behavior was one of the theories involved in conceptual framework that helped shape the the study and instrument design for data collection. For the secondary analysis, we used the IM of the TPB in order to create a structure from which to classify and view factors influencing BF. Therefore in the data we identified the following main categories: BF intentions and actual BF practices, behavioral beliefs, control beliefs, and normative beliefs. Taking other criteria of the IM into consideration, we categorized additional social influences on BF, including the family structure and influence (focused on the mother’s close female contacts like grandmother and mother-in-law), the father’s influence, and influence from healthcare providers. The definitions of all the categories we used for analyzing the data are as follows:

Intentions and practices: A participant’s reported intention or action with respect to BF.

Behavioral beliefs: The individual’s reported understanding and opinions about BF and its outcomes.

Control beliefs: The perceived ability to breastfeed, perceived self-efficacy, and perceived barriers and facilitators to BF.

Normative beliefs: There are differing definitions of norms in the literature. In the IM, normative beliefs include descriptive and injunctive norms, which we did not expound upon for this paper. We used a combination of definitions from Ajzen et al and Lapinski et al. [18,30]; what the individual perceived are the social pressures for what is acceptable or not acceptable BF behavior. Social norms refer to the collective normative beliefs among the study population.

Family Structure and Influence: Mention of the close or extended family structure of the mother, and how this was reported to influence BF behavior.

Influence of Father: Any mention of the father’s influence on BF.

Influence of Healthcare Providers: Any mention of influence or advice from clinics or healthcare providers.

### Data analysis

The quantitative data analysis was primarily descriptive, assessing percentages or means and standard deviations for survey questions. The analysis was conducted with STATA 12. For the secondary analysis of the qualitative data, two analysts used a thematic analysis approach (TMS and MAVB) using the software MAXQDA 12 [31]. We each first familiarized ourselves with the transcripts, and then TMS applied the categories from the conceptual framework to all corresponding narrative segments in the data. We divided some of the categories based on infant age (i.e., first 6 months after birth; after 6 months) to better understand BF practices and beliefs within the framework of WHO recommendations. TMS then looked for concepts within the categories, categorized the concepts among the narrative segments and analyzed these segments for emergent themes relating to BF. MAVB gave input and helped refine the concepts and themes in the final analysis. We established emergent themes based on the frequency and depth they were discussed in the qualitative data, and then used triangulation between the datasets for comparison and validation [32].

## Results

**Fig 2:**
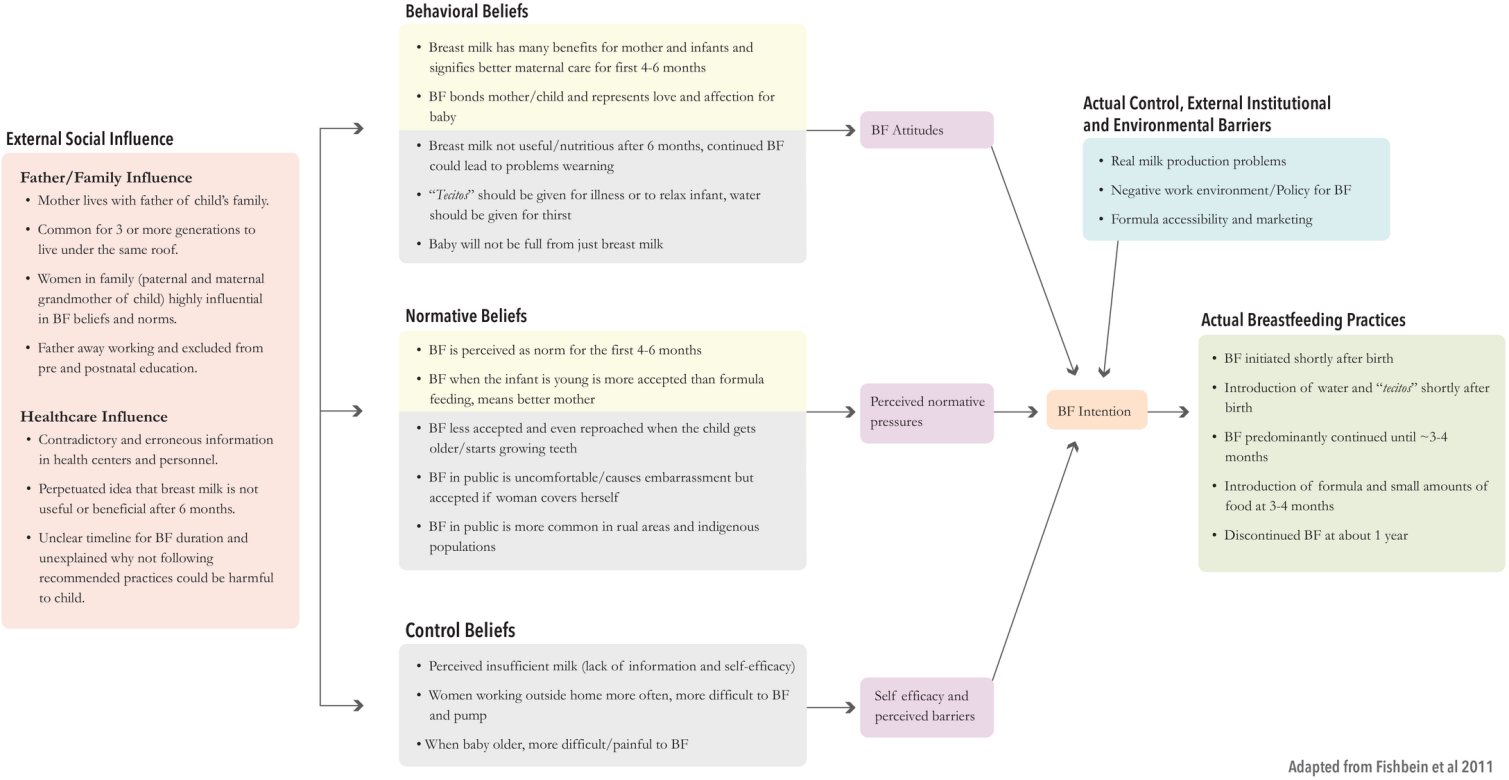
Integrated model of key findings.

A framework of our key findings using the IM of the TPB is shown in Fig 2.

A summary of basic demographics of participants and instruments from the original qualitative research are shown in Table 1. We did not find any significant difference between urban and rural areas in either dataset. This could be due to the similar lifestyles and sociodemographics shared between participants. *Prospera* beneficiaries share similar characteristics in that the program is targeted to those in the most extreme poverty in Mexico [33]. Typically fathers went to work in rural agriculture as field laborers or migrated for work regardless of urban or rural residence. The mothers reported themselves as caretakers of the home and not employed in the formal work sector. Six of the mothers and 13 of the community leaders said they additionally worked in informal jobs such as selling food on the street or domestic work (cleaning houses). The quantitative survey did not ask about work status of the mothers because it is the norm that *Prospera* beneficiaries are not employed in the formal work sector. The categories, concepts, and example quotes from the qualitative data, verbatim from the narrative segments to best represent the concept, are shown in Table 2. The socio-demographic characteristics for the quantitative data found in Table 3, and Table 4 shows key findings from the survey. The supplementary Tables 1–4 have more complete quantitative findings.

**Table 1:** Qualitative instrument and informant demographics.

Instrument		Focus groups—mothers	Focus groups—community leaders	In-depth interviews—Fathers
Total sample number (N)		50	44	10
Average age and range in years		31 (18–39)	41 (21–87)	35 (30–40)
Average number of children and range		6 (4–9)	6 (2–11)	3 (2–5)
Education (percent and n)				
	None	2 (1)		
	Primary (age 6–12)	50 (25)		60 (6)
	Secondary (age 12–15)	28 (14)		30 (3)
	Preparatory (age 15–18)	2 (1)		0
	University (age 18–23)	8 (4)		10 (1)
	Didn't answer	10 (5)		0
Work				*
	Amas de la casa (housewife)	50	44	
	Additional work (domestic service, street vendor, factory work)	6	13	

* The fathers reported working as field laborers, one a security guard for Coca-cola, in plumbing and masonry, and in "odd jobs"

**Table 2:** Categories, concepts, and exemplary quotes.

Category & Concept	Concept Description	Quotes
**Breast-Feeding Practices**	** **	
First 6 months—probaditas	BF first 6 months and introduction of "*probaditas*"	“Well at no later than 3 months, here we're used to making *canelita* [cinnamon tea infusion] or a *molotito* from corn dough [to give the baby]." **Mothers, Rural Oax.**
		I: “At 4 months, at three, four months you’ve already begun to give *probaditas*, because you say they stay hungry? Or why do you give it to them?” P: “Because they have to start getting used to eating other things… and their body is changing so it requires more things that breast milk can’t give them, they already need more vitamins, and, like from fruit and vegetables.” **Mothers, Rural Qro.**
First 6 months–water/teas	Introduction of water for thirst and "tecito" for illness after recently born	I: “And during the first 3 months you give them some water, *atole*, or other liquids or nothing nothing nothing?” P1: “No” I: “And when they’re colicky, what do you do?” P: “*Tecito*.” P2: “I give tea [meaning herbal infusion].” **Mothers, Rural Oax.**
		I: “When do you start giving water, or *tecitos*?” P: “All the time from when they’re very young.” I: “From very young, what age?” P: “From when they’re born.” **Mothers, Rural Qro.**
		I: “But then I could tell you, that’s not giving only breast milk, you’re also giving water, or is it different giving water?” P: “Uh-huh, well it’s because they sweat, that is why they get dehydrated also, because sometimes it’s very… it’s very hot and you cover them, you’re wriggling them, and they get really thirsty. And since the milk is luke-warm, they feel hotter, so you give them a little water. So they… so they lower their temperature.” **Mothers, Urban Oax.**
Beyond 6 months–Breastfeeding for 8 months—year	Discontinuation of BF after about 8 months/1y	I: “So, what is the reason you decide to quit breastfeeding?” P: “Well, I breastfed until, well, until they were already eating.” **Mothers, Rural Oax.**
Beyond 6 months–Practices to encourage weaning		P: “There are people that I see that put chili, they put sábila (Aloe Vera), they put a lot of things so that the child stops [breastfeeding] and gets scared.” **Mothers, Urban Oax.**
**Behavioral Beliefs**	** **	
First 6 Months	Breast milk is the best and healthiest for the first 4–6 months	I: “When they’re born what do you give them?” P1: “Breast.” P2: “Breast-milk.” I: “Breast milk? Why do you give it to them?” P1: “Because it’s the best.” P2: “Because it has defenses.” I: “And that’s the best? Why is that the best? You spoke of defenses… “P2: “Well yeah, because it protects from a lot of illnesses, and helps the baby improve its defenses, because when they are born they come without many defenses, so with breast milk it’s what helps them.” **Mothers, Urban Oax.**
	Breastfeeding makes mothers feel that they are bonding and caring for the baby.	I: “How do you feel, doing it [breastfeeding]?” P1: “It feels nice, it feels nice, you caress him.” P2: “It feels like you’re closer to him.” P1: “Yeah, with a lot of love.” **Mothers, Urban Oax.**
Beyond six months	Breast-milk not useful/nutritious after 6 months	I: “Uh, that’s what they tell you? P1: “Yes, that breast milk, it doesn’t serve anymore.” P2: “It no longer nourishes them.” P1: “It doesn’t have vitamins anymore, it no longer nourishes them, so you have to quit giving it to them the breast… Milk, *atolito*, whatever you could give them.” **Community Leaders, Urban Oax.**
	Continued breastfeeding could lead to problems weaning	P1: Also if you give them [breast milk] up to 2 years, later the child doesn’t want to eat.” P2: There are various women that told us that, that the child will want only breast.” **Community Leaders, Rural Oax.**
	"Tecitos" for illness passed down over generations	P1: “I, almost always was used to, always when my children were babies, after their bath, to give them their *tecito*, and then I give [breastlmilk] to them so they sleep a little, it was… my mother told us, that they gave them a *tecito*, and that was the way I used to do it when they were little.” **Mothers, Rural Qro.**
**Control Beliefs**		
Perceived Insufficient Milk	Principal reason for not breast-feeding was perceived insufficient milk and that baby cannot get full on just breast milk.	P1: “She was stuck to my breast, and was wanting more, more milk.” I: “So you were thinking that it was because she wasn’t eating enough milk? Or because you didn’t have enough milk?” P1: “Well, both of those.” I: “You guys think that with nothing more than pure breast milk they won’t get full?” P1: “No.” P2: “No.” P3: “No.” I: “No? Why do you say that?” P1: “Because when one doesn’t have a lot of milk the baby cries.” **Mothers, Urban Oax.**
Work	Women are more often working outside the home, in addition to their work in the home, percieved as a barrier	P: “Before, before the woman didn’t go out to work, the generation is changing because before the person that supported the home was the man, and now it’s both and well now from what I understand it’s both, well because now it’s not enough, so you have to leave the house to help them a little.” **Community Leaders, Urban Qro.**
		I: “Why didn’t they want to breastfeed?” P: “Because she was working.” **Mothers, Urban Oax.**
Breast-Milk Production	What you eat affects the quality and quantity of the milk you produce	I: “There are women that only have a little milk, do they have certain characteristics these women to those that have a lot of milk?” P: “I say that it depends a lot also on your diet. My doctor told me that you should drink more water, if you drink milk the milk will come, if you drink broth it will come, and if you eat fruit and vegetables it will also come, because you yourself are making sure your body has a lot of water, so the milk is going to come.” **Mothers, Urban Qro.**
Physical/Emotional Issues	BF too difficult/painful when baby starts growing teeth, or start grabbing or suctioning harder. More tiring when baby is older	I: “So, are there other reasons why the time comes when you stop breastfeeding?” P1: “Because also as they are growing they suck more, and like they hurt.” I: “So, it’s like it hurts more? Why?” P1: “It burns the chest.” P2: “It burns the skin.” **Mothers, Urban Oax.**
**Normative Beliefs**	** **	
Breastfeeding	Mothers feel that they are seen as better mothers if they breast-feed when the infant is young	I: “What type of, of perception could you have about women that breastfeed? What do you say about women who breastfeed?” P: “Well that we’re good mothers, because besides breastfeeding they give affection by embracing them.” **Community Leaders, Urban Oax.**
	Breastfeed for the first 6 months–year but not longer	I: “Those women that breastfeed the baby when it’s little, let’s say 6, 8 months, because that’s almost everyone, no? How do you see them, as something normal or not normal or how do you see them?” P1: “Something normal.” P2: “Normal.” **Mothers, Rural Oax.**
		I: “How do you see the mothers that breastfeed until 2 years?” P: “Well, we criticize them.” **Mothers, Urban Qro.**
	When the child gets older/starts growing teeth, breastfeeding becomes less accepted and is even reproached	I: “With an older baby, how do you see a woman that breastfeeds, a bigger baby?” P1: “What happens is that it already seems more, well it already looks ugly, one says ‘oops’. Or it embarrasses you, well for me I was embarrassed that [my baby] told me ‘I want, I want to eat,’ yes, I felt ashamed.” **Community Leaders, Rural Qro.**
Breastfeeding in Public	Breastfeeding in public socially uncomfortable but accepted if woman covers herself. Less accepted as baby is older. Looked down on in as something for poor, rural, and indigenous women	I: “How is the issue of breastfeeding in public? How do you do it?” P1: “Well one covers up, if one doesn’t wear anything.” P2: “Try that men are not present, it’s embarrassing.” P1: “Even covered, it’s embarrassing.” I: “So you don’t feel very comfortable breastfeeding in the street?” P1: “Even less in the hot season, the covered baby starts to pull at the blanket.” I: “And how do you see the women that breastfeed in the street?” P1: “It’s a necessity because the babies are crying.” P2: “I see it as normal, but there are perverted people.” P3: “The people that breastfeed in the street are the *Marías*, the indigenous women that carry them tied in their shawl. That’s what I’ve seen.” **Mothers, Urban Qro.**
		P1: “You cover [the baby].” P2: “You have to cover to breastfeed the baby, why? Because the man *nomás le echa la luz* [meaning the man stares at the breasts of a woman] **Community Leaders, Rural Oax.**
Structure and Influence of family	Norm is that mothers goes to live with father's family. Common for 3 generations to live under the same roof	I: “Where do you live?” P1: “Well, the mother-in-law’s house. With the mother of the muchacho [young boy].” I: “Uh-huh, it’s always that way?” P2: “Some yes, others go directly to their house.” I: “Uh-huh, but in general you guys all lived like that?” P1: “Yes.” **Mothers, Rural Oax.**
	Family members (especially women) highly influential in BF beliefs and norms	I: “You’ve spoken to me about various people who have given you recommendations… of mother-in-laws, mothers, nutritionists. How do you decide whom to listen to? Do they tell you the same things?” P1: “No. I look for opinions from my mom, my mother-in-law, and my sister, because they have more experience and based on that, those that are more correct about a single issue, is who I listen to.” **Mothers, Urban Oax.**
		P1: “The whole state of Oaxaca is used that the woman is, now almost, the mother and father for the children.” P2: “Before yes, right? They were used to the husband working and the woman was taking care of the home, it was nothing more than her home.” P3: “Now, no.” P2: “Unfortunately with the budget they bring, the wages they bring, it’s not enough for anything, so she, she abandons a little the home.” **Community Leaders, Urban Oax.**
Influence of Father	Different roles between the mothers and father; feeling that may be changing Fathers not a part of pre or post-natal care at health centers.	I: “Do they give you information in the health center?” P: “No.” I: “Why do you think they don’t give it to you?” P: Well I don’t know, now, well it’s always women that receive more information, in other words the housewives.” I: “Why do you think they give the information to them?” P: “In this case, because they have the program [*Prospera*], but there isn’t a program, for example for a person, call it a man, no, no there isn’t.” I: “Should there be do you think?” P: “I think it should exist…. Well yeah, for example the women receive information on how to take care of the children, illnesses, malnutrition, or whatever… and I, although I haven’t gone to the talks, logically we have to know it.” **Father, Urban Qto.**
		P: “Yes, well because the father logically says, well I don’t have time, I have to go work. But of course, they are a couple, so they should take [the men] into consideration so that they’re equal.” **Father, Urban Oax.**
Influence of Healthcare providers	Contradictory and erroneous information from health centers and personnel Breast-milk is not useful or beneficial after 6 months.	P1: “Well before they had told us [to breast feed] until 8 months, but recently, it hasn’t been long, we had a meeting and they told us until 2 years here in the health center.” P2: “They told me until 5 months.” **Mothers, Rural Oax.**
		P1: “But they say that it’s not useful, it’s not the same. I: “Who says that?” P1: The pediatrician.” P2: “The pediatrician.” I: “The pediatrician says it’s [breast milk] no longer useful?” P1: “That it’s now pure water.” P2: “That you can give it until you want, but now it doesn’t have the same vitamins.” **Mothers, Rural Qto.**

For semantic accuracy, a native English and fluent Spanish-speaker (TMS) and a native Spanish and fluent English-Speaker (MAVB) worked together to translate the quotes.

“I” indicates “Interviewer” and “P” indicates “Participant”–the P is numbered in some quotes to indicate different participants speaking.

**Table 3:** Quantitative socio-demographic characteristics.

Variable	Percentage or mean and SD (n = 321)
Maternal Status	
Currently pregnant	33.33
Mother of child <6months	33.33
Mother of child <12months	24.3
Mother of child 12–24 months	9.03
Average Age	25±6.12
Area	
Urban	41.74
Rural	58.26
*Oportunidades* [*Prospera]* Beneficiary	
Yes	27.81*
No	72.19
What is your civil status	
Single	11.84
Married	41.74
Domestic Partnership	45.17
Widow	0.31
Separated	0.93
Divorced	0
Do you speak an indigenous language	
Yes	8.46
No	91.54
What was the last completed grade or year in school?	
None	1.87
Kindergarten/Preschool	0.93
Primary School	25.23
Secondary School	47.04
Technical School	3.12
Preparatory School	14.33
University	4.67
Other	2.8
Who do you live with in your home?	
Partner/Spouse	84.74
Child/Children	83.18
Father or Mother	25
Grandfather	0.62
Grandmother	1.25
Brother(s)	14.02
Sister(s)	11.53
Brother/Sister-in-law	13.71
Son/Daugher-in-law	0.31
Niece/Nephew	4.98
Cousin	1.56
Father-in-law	16.2
Mother-in-law	20.56
Aunt/Uncle	1.87
Adopted child	0.31

*The national percentage of *Prospera* beneficiaries is 21% (http://cuentame.inegi.org.mx/poblacion/habitantes.aspx?tema=P)

Data are mean ± SD values or percentage (n) per participant

**Table 4:** Quantitative key findings.

Variable	Percentage or mean and SD (n = 321)
**Breastfeeding Intention**	
During your pregnancy are you planning (did you plan) to only breastfeed your baby, without giving any other liquids or milk or small bites of food?	
Yes	70.22
No	29.78
For how long are you planning (did you plan) to only breastfeed your baby, without giving any other liquids or milk or small bites of food?	5.9m±3m
**Behavioral Beliefs**	
What do you think about giving only breast milk to your baby for 6 months?	
Agree	71.96
Disagree	22.74
Don't know	5.3
What does only breastfeeding your baby mean to you?	
To breast feed, does not include water or other liquids	57.79
To breast feed, without giving other milk	22.08
To breast feed plus water/tea infusion	6.82
To breast feed and give formula	3.25
To breast feed and occasionally give small bites	1.95
Other	8.12
**Control Beliefs**	
In your opinion, what would be the principal difficulties in giving only breast milk (without water, any other liquid or milk, or small bites of food) for the first 6 months?	
Nothing/no reason	2.8
The baby will still be hungry, breast milk doesn’t fill them	6.23
Not having sufficient milk	37.69
The milk is not nutritious	3.74
Painful breasts	2.8
Lack of time	2.49
Lack of experience/practice	2.8
The baby doesn't want it or decided not to take it from the beginning	1.87
Have to go to work	6.54
Worried it will deform the breasts or roughly handle them	2.49
The nipples were not well-formed	4.67
Other	17.13
Don't know	8.72
**Normative Beliefs and External Influences**	
Do you know anyone (family member or acquaintance) who only breastfed or is only breastfeeding (without giving water, nor other liquids, milk, or small bites of food) for the first 6 months of the baby’s life?	
Yes	30.84
No	69.16
What for you would be the reason most important this person breastfed or is only breastfeeding (without giving water, nor other liquids, milk, or small bites of food) for the first 6 months of the baby’s life?	
Their doctor recommended it	10.1
Their mother recommended it	23.23
Their mother-in-law recommended it	4.04
Because it was best for the baby	0
Because they had previous experience with their other children	15.15
Because she doesn´t have money to buy other milk	6.06
Because she is a good mother	2.02
Because she is lazy and didn't want to prepare other foods	6.06
Because her work permitted her time	1.01
Because the baby accepted it	4.04
Because she's young and healthy	1.01
Because she had plenty milk	9.09
Because she has partner/spouse	1.01
Other	11.11
Don't know	6.06
If you have any questions about how to feed your baby only with breast milk (without giving water, nor other liquids, milk, or small bites of food), where do you look for advice/help?	6.06
Books or magazines	1.25
Doctor	63.75
Nurse	16.56
Health promotor	3.44
Oportunidades [*Prospera*] Vocal	0.62
Friends or family	10.94
Mother	42.19
Mother-in-law	20.31
Partner/spouse	1.25
Television/radio	0.31
Internet	5.94
Specialist (Pediatritian)	5.31
Other	7.81

### Breastfeeding intentions & practices

#### First 6 months after birth

The most common practice reported in all focus groups was early initiation of BF within a few hours after birth and predominantly BF until around 3–4 months. The introduction of water for thirst and "*tecitos*" (hot tea) for colic, minor illness, or to relax the baby was customary and initiated most commonly from around birth to two months after birth and continued thereafter. In the survey, 70% of women said they were planning to breastfeed their baby, without giving any other food or liquid, and the average length they planned to give only breast milk was 5.9 months. In focus groups with mothers, when asked if they fed their baby only breast milk for the first 6 months, women typically said yes, but then when asked how they treated illness or thirst for infants, the women said with *tecitos* or water. Additionally, many reported giving “*probaditas*” or little bites of food, beginning at about 3–4 months. Mothers also reported giving formula early after birth if they felt they were not producing enough milk. In the quantitative data, 27% of women were planning to give formula to their baby, and the main reason was because with only breast milk the baby would not be full (17%) or because the mother had to return to work (15%).

#### After 6 months

In focus groups, women women reported BF about a year, although the duration of BF varied among the participants. Some women actively tried to prevent BF after about 8 months to a year for fear the baby not want to stop BF and therefore not transition to eating other food. In order to wean, some mentioned putting chili on their nipple to repel the baby. However, in the survey the average length that women reported intending to breastfeed was 17 months, and the average length they were planning to give formula was 22 months.

### Breastfeeding behavioral beliefs

#### First 6 months after birth

Mothers and community leaders said they wanted to do what was best for the baby, and their decisions were based on perceived healthiest behaviors. In general, all participants, including fathers, believed that breast milk was the best and healthiest for the baby, and that breast milk had nutritional properties that help the infant’s immune system. Participants also mentioned benefits for the mother, such as prevention of breast cancer. Also frequently mentioned was that BF bonds the mother and child, represents the mothers’ love and affection for the baby. Conversely, however, women and community leaders mentioned the fear that the infant would not be full with breast milk alone, and that water was necessary in addition to breast milk for thirst. In the survey, the majority of participants agreed with giving only breast milk to their infant for the first 6 months (72%) and said it was because it’s best for the baby (60%) and helps the immune system of the baby (17%). Of those that disagreed with giving only breast milk for the first 6 months, more than 40% said the baby would not be full from breast milk alone and 12% said it was because the baby would remain thirsty. Nearly 58% of women correctly defined EBF as giving only breast milk without any other foods or liquids, but 22% thought that EBF was giving breast milk but not other types of milk. Only 6.8% thought EBF was breast milk plus water and tea.

#### After 6 months

In the focus groups and interviews, there was the common belief that breast milk was not useful or nutritious after 6 months, which the women reported being told by healthcare providers. Also consistent was fear that if the mother continued to breastfeed up to 2 years, the baby would not want to eat other food and would end up losing weight and being too dependent on the breast.

### Breastfeeding control beliefs

#### Perceived insufficient milk

A principal perceived barrier to BF discussed in the focus groups with both mothers and community leaders was the inability to produce sufficient milk to feed the baby. This barrier was seen as an accepted reason for other mothers to give formula instead of breastfeed. Similarly in the survey, when asked what the principal difficulty would be for only giving breast milk the first 6 months, the most prevalent cause cited was not having sufficient milk (38%).

#### Physical and emotional discomfort

Both mothers and community leaders in focus groups said that when the baby started growing teeth or started grabbing or suctioning harder, they were more likely to stop BF because of the discomfort or pain. In the survey, when asked what the principal reason would be to stop BF, the main reasons were cultural illnesses for fear or anger, *susto* or *coraje* (36%), and the second reason was that the baby would not be well fed (19%).

#### Breast milk production

Even among fathers, there was a common belief that what the mother eats affects the quality and quantity of the milk she produces. There were certain foods and drinks, such as *caldo* (broth) and *atole* (traditional hot drink) that were thought to promote milk production, and other foods such as some cold foods, spicy food thought to impede milk production. Many participants said that if the mother had problems producing enough milk, it was due to her diet. The survey suggested similar perceptions; the main factor a woman said she would need in order to be able to breastfeed exclusively for the first 6 months was to eat well, including fruits and vegetables (54%). The next reason was to drink enough water (11%).

#### Work

Some mothers said they were working informally outside the home (selling food on the street, domestic work etc) as well as being the primary homemaker. Although most are able to bring their baby with them, they reported that this extra burden, in addition to housework and childcare, interfered with BF, especially continued BF when the baby was older. In the survey, when asked why the mother planned to (or did) give formula or other milk (different from breast milk) to their baby 15% said it was because of work, 17% said with only breast milk the baby will not be full, and 32% said “other”.

### Breastfeeding normative beliefs

In focus groups, mothers felt that they were seen as better mothers if they breastfed when the infant was young. In the survey when asked about attitudes towards mothers who do not give only breast milk for the first 6 months, the most prominant reason was they thought it is because the mother does not take care of her baby (20%). In the focus groups with both mothers and community leaders, however, when the child was older, specifically when they started growing teeth, BF was less accepted. In general, women saw BF in public as socially uncomfortable but acceptable as long as the woman covered herself. There were some references from mothers and community leaders about women who breastfed in public being poor, rural, and indigenous women, and some participants reported feeling as though would be judged negatively for doing so. BF in public was less accepted or not accepted at all if the baby was older (more than a year, or had already started growing teeth). Conversely, a few fathers spoke of BF in public and they all said they saw it as normal.

### External influences

#### Family structure and influence

Most often reported was that the mother would live in the house of her husband's family, especially in rural areas. It was common for at least 3 generations of the mother or father of young children to live under the same roof, and often with extended family as well (cousins, nieces/nephews). In focus groups the mothers said they were often around other women who had gone through raising children or who also had small children, and therefore had a close community of women with current or previous BF experience that influenced beliefs and practices. In the survey, over 60% of the women said their close family members would agree with giving only breast milk for the first 6 months, but of those who said their family member would not agree, the main reason was that they would think the baby would still be hungry (34.92%) or that the baby should also be given water and tea (29.37%).

#### Influence of father

Almost all the participants, including fathers, spoke about distinct roles between the mother and father; the mother’s role was to care for the children and home, and the father left home to work. Fathers were often gone all week and only present some of the weekend, or potentially gone for months at a time if they migrated for work. Men also reported minimal participation in the pre and post-natal care of their partner and infant. Some fathers reported feeling excluded from information or workshops offered at the health centers.

#### Influence of healthcare providers

In all focus groups, participants reported contradictory and sometimes erroneous information at health centers. Women said that healthcare providers recommended BF for the first months (although the duration varied), but that the breast milk would no longer be useful after several months to a year, it is like “water” or “blood” and loses its nutritional benefits. Advice on duration of BF and its benefits varied greatly, as well as the duration and benefits of continued BF. In the quantitative data, influence from health centers was reportedly high. Women said that the place they were most often spoken to about BF was the health clinic (66.36%) and that the person who most often spoke to them was the doctor (39.73%), nurse (26.94%), then Health Promoter (13.24%). When asked about which person would be most influential in the care of the baby, the women said primarily their doctor (52.17%) and next their mother (19.13%). When asked whom they go to for advice or to whom they pay most attention, similarly most they also said doctor (50%) and mother (23.75%).

## Discussion

Our study found that behavioral and normative beliefs and BF practices among our study populations supported BF, but were not aligned with recommmended practices. In general, women breastfed, but not exclusively, for about the first 6 months, and sporadic BF after 6 months was normal to about 8 months or a year. Our study gave new insight into the reasons why and in what way recommended practices are not followed, which can help illuminate how to improve future promotional efforts in Mexico and in other parts of the world.

It was clear in both quantitative and qualitative data that there were two principal reasons for not EBF for the first 6 months. One reason was the belief that babies will not be full with breast milk alone, the concern manifesting most commonly a few months after birth. As a consequence, infants were given formula and small bites of food in addition to breast milk. The other reason was the belief that infants need water in addition to breast milk to properly hydrate, and the use of teas for treating colic, beginning soon after birth. Other studies have shown similar beliefs and practices among low-resource populations, who, soon after birth, similarly supplemented breast milk with water for thirst and other foods or formula for fear the baby would remain hungry or not be sufficiently fed [34,35]. Most likely the population believes that is how one should feed an infant and does not distinguish the difference and definition of exclusive versus non-EBF. For instance, nearly 60% of women from the survey correctly defined EBF while only about 7% thought that EBF should also include water and tea. In the qualitative data, the women said they only fed their baby breast milk for the first 6 months. Yet they reported also giving water and teas shortly after birth and small amounts of food as early as 3 months, indicating that although they may be able to correctly define EBF, they do not apply the definition to practice. Within the context of the IM of the TPB, in order to better influence BF practices efforts should be made to underscore the positive behavioral beliefs and to counter behavioral beliefs that contradict exclusive BF. In other words, efforts have to distinguish between giving breast milk and giving *only* breast milk for the first 6 months. Many interventions state BF recommendations without explaining why; they need to emphasize that water and small bites of food before 6 months could result in water intoxication in infants, more risk of diarrheal diseases, the possible reduction in breast milk supply with the supplementation of non-breast milk liquids and solids, and the fact that introducing water, teas, and solid foods replaces important nutrients and calories in breast milk and could cause serious weight loss and illness in the infant [36].

Moreover, the acceptability of BF decreased as the baby got older, until it seemed scornful for a mother to breastfeed a toddler. One of the substantial behavioral beliefs we identified was the idea that breast milk is neither useful nor nutritious past about 4–6 months or a year. This belief is also probably instrumental in the early discontinuation of BF, and could also contribute to social unacceptability of BF when the baby is older. While there was preference to breastfeed over bottle-feed an infant, as it was seen as more maternal and loving to the baby, continued BF the baby “once it has grown teeth” was derided. This belief was perpetuated in the clinics, among providers, and also in communities, so future efforts need to address why breast milk remains beneficial up to 2 years after birth.

The percentage of participants in agreement with giving only breast milk for the first 6 months was high (71%). Additionally, the qualitative data suggested knowledge of healthy benefits for infant and mother, and the association between BF and better maternal care also indicated positive normative beliefs. Yet these norms were in striking contrast with actual BF rates as reported in 2012, which was nationally low and in rural and indigenous populations had dropped from 1999 nearly 20 percentage points [13]. The difference could be that the population does not understand the distinction between BF and EBF in practice, even though many were able to properly define it in the survey. Another possibility is that the gap between attitudes and behavior is due to the many barriers that continue to impede healthy BF practices, despite the positive norms.

For example, our study found similar BF obstacles to earlier research not only in Mexico but in many other populations, most notably that the primary individual-level barrier to BF was the perception that the mother cannot produce enough milk and that the infant will remain hungry with only breast milk [37–42]. One explanation Bonvecchio et al mentions is that there is little education on infant growth spurts, which may cause a woman to feel she is not producing enough even though the body eventually compensates for increased consumption [37]. Further evidence has also linked decreased BF rates and perceived insufficient milk, in particular among low-resource populations, with Westernization and urbanization, the symbolization and sexualization of breasts, more women in the workforce, and especially effective formula marketing and easy access [43,44]. These reasons could be applicable to the communities in this study. When addressing control beliefs, as shown in the IM of the TPB, interventions need to address self-efficacy as well as actual control problems with BF. This means having resources available when women face difficulty BF, and strong social support to help them problem-solve. Additionally, Guerrero et al and Bueno-Gutierrez et al., both mention the importance of emphasizing cultural traditional values as an important part of BF promotion, as modern life can be seen as a cause for obstructing recommended practices [45,46]. Our research also supports highlighting the traditional perception that BF is normal and natural, and changing social norms to circle back to these beliefs.

The disapproving attitude towards BF in public, for example as one participant said that BF in public is for “*the “*marías,*” the indigenous women who are around and have them [the babies] tied up with a* reboso *[sling]*,” could indicate some remnants of Westernization; that is, the idea that BF is still seen as something “uncivilized” [43]. Studies among low-resource populations in other areas have found similar apprehension and anxiety about disapproval of BF in public [47,48]. As Victoria et al point out, feelings of embarrassment are commonly reported with BF in public, whereas bottle-feeding rarely receives negative attention [2]. The few fathers that spoke on the issue seemed to indicate a different perspective, that they view it as normal, which could imply different normative beliefs. However, the issue was not discussed sufficiently with the fathers in the interviews, and warrants further investigation. From the lens of the IM of the TPB, it would be nearly impossible to change individual behavior without addressing the influence of external contacts to the mother. Future efforts need to focus on reversing normative beliefs that BF an older child is unnecessary and shameful, and that BF in public is something that is only done in poor rural areas and should be hidden. A 2000 Cochrane review found that mass media, and in particular television commercials, helped increase positive attitudes and in turn affected BF initiation rates [49]. Future promotional efforts should consist of a multi-pronged approach that includes mass media to help shape social norms.

Since the idea that the baby will not be full on breast milk alone was such a common belief in the population, and the perceived inability to produce milk was a dominant barrier, more emphasis needs to be placed on both alleviating this worry, educating about milk production, and increasing self-efficacy and external support for mothers. It was clear from the data that both healthcare providers and women in the mother’s family structure (particularly the maternal and paternal grandmothers of the infant) were influential in BF practices. What was also apparent was the lack of the father’s role in pre-natal and early care of the child. The fathers mentioned that they were not often given information or included at the health centers. Yet given the literature that demonstrates the importance of the father’s influence [46,50] as well as the sentiment among participants that father’s involvement may be changing, future efforts in Mexico need to be more inclusive of fathers.

There were some limitations to our study, one of which was the use of only pre-existing data. Because it was a secondary analysis, it was not possible to tailor survey or interview questions to more directly address normative beliefs, although that was one of the objectives in the original data collection. Additionally, the data came from 2 Mexican states and the quantitative data included a sample size chosen by convenience. However, the states and areas were chosen because they were representative of bigger populations, and the quantitative data had representation from many localities. *Prospera* beneficiaries typically do not work in the formal work sector, which may bias results, since work is a significant barrier to BF in many populations [51–54]. Yet it is important to point out that work did not factor in to the principal reasons why our population was not practicing EBF or continuing to breastfeed up to two years. Because the mothers we spoke to work mainly in the home and informal jobs, they were most often able to keep their baby close, but there were still other influences impeding recommended BF practices. This insight could also be useful for generalizing to other low-resource populations with similar characteristics. Lastly, since participant recruitment for the focus groups was through membership of the federal program *Prospera*, it did not include many younger women, which may have biased the results, as younger mothers most often have lower BF rates [55].

Our study, nonetheless, had considerable advantages. This study was unique in that we investigated various BF influences and particularly normative beliefs among low-resource communities in Mexico, of which there has been little previous research. Our study also included interviews with fathers, whose voice has not generally been included in previous research. Additionally, the variety of mixed methods techniques we used to collect and analyze the data strengthened our results. These results allowed for a more profound understanding of the reasons behind BF practices and beliefs and will contribute significantly to improving future promotional efforts.

## Conclusions

What most greatly motivates BF intentions and behavior among the study population was what was considered the best and healthiest care for their baby, and these beliefs appeared to be significantly shaped and influenced by the behavioral and normative beliefs perceived from the family and community, as well as from healthcare providers. While the norms shed a positive light on BF, they did not support EBF for the first six months or continued BF up to 2 years. Future strategies to promote BF among low-resource communities need to specifically address the difference between BF and EBF, and why EBF is superior. Also important is to better understand the causes of perceived insufficient milk and how to counteract them, and to promote self-efficacy for and knowledge about milk production. Efforts should include different instruments such as mass media to inform social norms, as well as improving knowledge and influence among healthcare providers as well as close family members, including fathers. BF promotion should try to reverse the practices and norms of giving water, tea, and small bites to infants before 6 months after birth, and improve the acceptability of BF in public, thereby empowering women to do so.

## Supporting information

S1 Table Breastfeeding intention or behavior. (DOCX)

S2 Table Breastfeeding behavioral beliefs. (DOCX)

S3 Table Breastfeeding control beliefs. (DOCX)

S4 Table Breastfeeding normative beliefs & external influences. (DOCX)

S1 Appendix EsIAN quantitative survey (note: the authors did not use all information from the survey, only the sections pertaining to the secondary analysis). (PDF)
