# Supplementary material for: Breastfeeding practices, beliefs, and social norms in low-resource communities in Mexico: Insights for how to improve future promotion strategies
Source: PLoS One. 2017 Jul 3;12(7):e0180185. doi: 10.1371/journal.pone.0180185 (PMC5495390; doi:10.1371/journal.pone.0180185)
Supplement: S1 Table — (DOCX) [file pone.0180185.s001.docx]

**Supplementary Table 1. Breastfeeding Intention or Behavior**

| Variable | Percentage or mean and SD (n=321) |
| --- | --- |
| During your pregnancy are you planning (did you plan) to only breastfeed your baby, without giving any other liquids or milk or small bites of food? |  |
| Yes | 70.22 |
| No | 29.78 |
| For how long are you planning (did you plan) to only breastfeed your baby, without giving any other liquids or milk or small bites of food? | 5.9m±3m |
|  |  |
| For how long are you planning (did you plan) to breastfeed along with other foods? | 17m±24m |
|  |  |
| During your pregnancy, are you planning (did you plan) to give formula or other milk to your baby? |  |
| Yes | 27.41 |
| No | 71.34 |
| During your pregnancy, up to what age are you planning (did you plan) to give formula? |  |
|  | 22±26 |
| Why do you plan (did you plan) to give other formula or other milk (different from breast milk) to your baby? |  |
| Because I work | 14.77 |
| Because it's best for baby | 10.23 |
| Because it feeds the baby | 12.5 |
| Because with only breastmilk it doesn't get full | 17.05 |
| Because formula fills him/her | 6.82 |
| Because it's easy to prepare | 0 |
| Because it was recommended by a nurse/doctor | 5.68 |
| other | 31.82 |
