# Supplementary material for: Breastfeeding practices, beliefs, and social norms in low-resource communities in Mexico: Insights for how to improve future promotion strategies
Source: PLoS One. 2017 Jul 3;12(7):e0180185. doi: 10.1371/journal.pone.0180185 (PMC5495390; doi:10.1371/journal.pone.0180185)
Supplement: S2 Table — (DOCX) [file pone.0180185.s002.docx]

**Supplementary Table 2. Breastfeeding Behavioral Beliefs**

| Variable | Percentage (n=321) |
| --- | --- |
| What do you think about giving only breast milk to your baby for 6 months? |  |
| Agree | 71.96 |
| Disagree | 22.74 |
| Don't know | 5.3 |
| Why do you agree with giving only breast milk for the first 6 months (without water nor any other liquid or milk or small bites of food)? |  |
| Because it's best for baby | 59.83 |
| Because it's sufficient and adequate | 3.85 |
| Because it's okay | 5.56 |
| Because it gives defenses against illnesses | 16.67 |
| Because you give love to the baby | 0.43 |
| Because it's cheap and practical | 0 |
| Because it is nutritious and helps the baby grow | 8.97 |
| Other | 2.99 |
| Don't know | 1.71 |
| For the above, what would be the most important for you? |  |
| Because it's best for baby | 45.73 |
| Because it's sufficient and adequate | 4.27 |
| Because it's okay | 5.98 |
| Because it gives defenses against illnesses | 24.36 |
| Because you give love to the baby | 0.43 |
| Because it's cheap and practical | 1.28 |
| Because it is nutritious and helps the baby grow | 13.25 |
| Other | 2.99 |
| Don't know | 1.71 |
| Why do you disagree with giving only breast milk for the first 6 months (without water nor any other liquid or milk or small bites of food)? |  |
| Because I don't have sufficient milk | 8.86 |
| Because the baby would remain hungry | 40.51 |
| Because the baby would be thirsty | 11.39 |
| Because the baby won't be fed well | 11.39 |
| Because the baby wouldn’t grow sufficiently | 5.06 |
| Because it's not okay | 1.27 |
| Because the mothers works | 2.53 |
| Because it's tiring to breastfeed | 0 |
| Other | 18.99 |
| What do you think are the benefits/advantages for the baby only receiving breast milk (without water, any other liquids, other milk, or small bites of food) for 6 months? |  |
| Gives love to baby | 3.75 |
| Helps give defenses against illnesses | 45.62 |
| It's cheap and practical | 1.56 |
| It's available everywhere | 1.88 |
| It's the first vaccine | 0.94 |
| To be healthy | 17.81 |
| To help the baby grow | 5.31 |
| It gives nutrients | 12.19 |
| To prevent chronic diseases in the future | 1.88 |
| It's normal/tradition | 0 |
| Other | 4.69 |
| Don't know | 4.38 |
| What do you think are the benefits/advantages for the mother only giving breast milk (without water, any other liquids, other milk, or small bites of food) to the baby for 6 months? |  |
| Its free (economical) | 22.43 |
| It's practical (available everywhere) | 14.64 |
| The mother loses weight | 10.9 |
| It helps the mother not become pregnant again (contraceptive) | 0.31 |
| It lowers postpartum bleeding | 0.31 |
| It lowers postpartum depression | 0 |
| It lowers the risk of breast cancer | 14.33 |
| It lowers the risk of uterine cancer | 0.31 |
| None/It's normal/tradition | 5.92 |
| Other | 7.48 |
| Don't know | 23.36 |
| What does only breastfeeding your baby mean to you? |  |
| To breast feed, does not include water or other liquids | 57.79 |
| To breast feed, without giving other milk | 22.08 |
| To breast feed plus water/tea infusion | 6.82 |
| To breast feed and give formula | 3.25 |
| To breast feed and occasionally give small bites | 1.95 |
| Other | 8.12 |
| Do you know or has anyone told you for how long it is recommended to only breast feed after the baby is born, without water, tea infusion, or other liquids? |  |
| Yes | 71.96% |
| No | 22.74% |
| Don’t know | 5.3% |
| How long after the birth of the baby have you been told or have you heard  you should only breastfeed (without water, tea infusion, or other liquids)? | 7.26±9.03 |
