# Supplementary material for: Breastfeeding practices, beliefs, and social norms in low-resource communities in Mexico: Insights for how to improve future promotion strategies
Source: PLoS One. 2017 Jul 3;12(7):e0180185. doi: 10.1371/journal.pone.0180185 (PMC5495390; doi:10.1371/journal.pone.0180185)
Supplement: S3 Table — (DOCX) [file pone.0180185.s003.docx]

**Supplementary Table 3. Breastfeeding Control Beliefs**

| Variable | Percentage (n=321) |
| --- | --- |
| What do you think you would need to be able to give only breast milk (without water, any other liquid or milk, or small bites of food) for the first 6 months? |  |
| Time | 6.54 |
| Someone to help take care of the other children | 0.31 |
| Someone to help with housework | 0 |
| Someone to teach me how | 2.8 |
| Eating well (including fruits and vegetables) | 54.21 |
| Drinking more water | 10.59 |
| Someone to explain to me how to pump | 0.93 |
| Nothing | 9.03 |
| That the baby accepted it | 2.49 |
| Other | 9.66 |
| Don't know | 3.43 |
| In your opinion, what would be the principal difficulties in giving only breast milk (without water, any other liquid or milk, or small bites of food) for the first 6 months? |  |
| Nothing/no reason | 2.8 |
| The baby will still be hungry, breast milk doesn’t fill them | 6.23 |
| Not having sufficient milk | 37.69 |
| The milk is not nutritious | 3.74 |
| Painful breasts | 2.8 |
| Lack of time | 2.49 |
| Lack of experience/practice | 2.8 |
| The baby doesn't want it or decided not to take it from the beginning | 1.87 |
| Have to go to work | 6.54 |
| Worried it will deform the breasts or roughly handle them | 2.49 |
| The nipples were not well-formed | 4.67 |
| Other | 17.13 |
| Don't know | 8.72 |
| What do you think are some of the main reasons women stop producing milk? |  |
| Taking contraceptives | 0.31 |
| Susto (being frightened) or Coraje (feeling mad) | 35.83 |
| The baby eats a lot and milk is finished | 0.93 |
| The baby isn't well fed | 18.69 |
| The woman doesn't drink enough *atole* or liquids | 7.17 |
| Other | 18.69 |
| Don't know | 18.38 |
| For above, what would be the most important reason for women to stop producing milk? |  |
| Taking contraceptives | 0.31 |
| Susto (being frightened) or Coraje (feeling mad) | 33.02 |
| The baby eats a lot and milk is finished | 0.62 |
| The baby isn't well fed | 19 |
| The woman doesn't drink enough *atole* or liquids | 8.72 |
| Other | 19.94 |
| Don't know | 18.38 |
| Do you think you could give (or would be able to give) only breast milk (without water, nor any other liquid or milk, or small bites of food) to the baby for 6 months? |  |
| Yes | 62.93 |
| No | 36.45 |
| Don't know | 0.62 |
| (If answered NO to above question) Why do you think you can´t give (or would not be able to give) only breast milk (without water, nor any other liquid or milk, or small bites of food) to the baby for 6 months? |  |
| Because I am underweight | 0.85 |
| Because I don't have enough milk | 17.09 |
| Because I work | 4.27 |
| Because I don't want to | 1.71 |
| Because the baby needs water | 36.75 |
| Because the baby needs tea infusions for colic | 1.71 |
| Because the baby isn't growing | 4.27 |
| Because the baby asks for other foods | 14.53 |
| Other | 17.95 |
| Don't know | 0.85 |
