# Supplementary material for: Breastfeeding practices, beliefs, and social norms in low-resource communities in Mexico: Insights for how to improve future promotion strategies
Source: PLoS One. 2017 Jul 3;12(7):e0180185. doi: 10.1371/journal.pone.0180185 (PMC5495390; doi:10.1371/journal.pone.0180185)
Supplement: S4 Table — (DOCX) [file pone.0180185.s004.docx]

**Supplementary Table 4. Breastfeeding Normative Beliefs & External Influences**

| Variable | Percentage (n=321) |
| --- | --- |
| During your pregnancy, did anyone speak to you about breastfeeding? |  |
| Yes | 68.54 |
| No | 31.46 |
| Where were you spoken to about breastfeeding? |  |
| Health Center | 66.36 |
| IMSS clinic | 9.55 |
| SSA Hospital | 3.18 |
| Midwife | 0 |
| Oportunidades [Prospera] workshop | 2.73 |
| Seguro Popular workshop | 0 |
| Private Clinic consultation | 4.09 |
| Other | 14.09 |
|  |  |
| During your pregnancy, who spoke with you about breastfeeding? |  |
| Doctor | 39.73 |
| Nurse | 26.94 |
| Health Promoter | 13.24 |
| Health Auxiliary | 2.28 |
| Oportunidades [Prospera] Vocal | 0.46 |
| Midwife | 0 |
| Mother | 8.68 |
| Mother in-law | 0.46 |
| Family member (sister, aunt, etc.) | 3.2 |
| Other | 5.02 |
| During your pregnancy, did anyone recommend giving formula (or bottle milk) to the baby? |  |
| Yes | 12.5 |
| No | 87.5 |
| During your pregnancy, where were you spoken to about giving formula to the baby? |  |
| Health Center | 25 |
| IMSS clinic | 0 |
| SSA Hospital | 2.5 |
| Midwife | 0 |
| Oportunidades [Prospera] workshop | 0 |
| Seguro Popular workshop | 0 |
| Private Clinic consultation | 12.5 |
| Other | 60 |
| During your pregnancy, who spoke with you about giving formula to the baby? |  |
| Doctor | 32.5 |
| Nurse | 15 |
| Health Promoter | 2.5 |
| Health Auxiliary | 0 |
| Oportunidades [Prospera] Vocal | 0 |
| Midwife | 0 |
| Mother | 12.1 |
| Mother0in0law | 2.5 |
| Family member (sister, aunt, etc.) | 27.5 |
| Other | 7.5 |
| Do you know anyone (family member or acquaintance) who only breastfed or is only breastfeeding (without giving water, nor other liquids, milk, or small bites of food) for the first 6 months of the baby’s life? |  |
| Yes | 30.84 |
| No | 69.16 |
| Why do you think this person breastfed or is only breastfeeding (without giving water, nor other liquids, milk, or small bites of food) for the first 6 months of the baby’s life? |  |
| The hospital or clinic oriented them | 12.12 |
| Their doctor recommended it | 23.23 |
| Their mother recommended it | 2.02 |
| Their mother-in-law recommended it | 0 |
| Because it was best for the baby | 15.15 |
| Because they had previous experience with their other children | 5.05 |
| Because she doesn´t have money to buy other milk | 2.02 |
| Because she is a good mother | 7.07 |
| Because she is lazy and didn't want to prepare other foods | 1.01 |
| Because her work permitted her time | 2.02 |
| Because the baby accepted it | 1.01 |
| Because she's young and healthy | 1.01 |
| Because she had plenty milk | 10.1 |
| Because she has partner/spouse | 1.01 |
| Other | 11.11 |
| Don't know | 6.06 |
| What for you would be the reason most important this person breastfed or is only breastfeeding (without giving water, nor other liquids, milk, or small bites of food) for the first 6 months of the baby’s life? |  |
| Their doctor recommended it | 10.1 |
| Their mother recommended it | 23.23 |
| Their mother-in-law recommended it | 4.04 |
| Because it was best for the baby | 0 |
| Because they had previous experience with their other children | 15.15 |
| Because she doesn´t have money to buy other milk | 6.06 |
| Because she is a good mother | 2.02 |
| Because she is lazy and didn't want to prepare other foods | 6.06 |
| Because her work permitted her time | 1.01 |
| Because the baby accepted it | 4.04 |
| Because she's young and healthy | 1.01 |
| Because she had plenty milk | 9.09 |
| Because she has partner/spouse | 1.01 |
| Other | 11.11 |
| Don't know | 6.06 |
| What is your opinion of this person breastfed or is only breastfeeding (without giving water, nor other liquids, milk, or small bites of food) for the first 6 months of the baby’s life? |  |
| Agree | 91.92 |
| Disagree | 7.07 |
| Don't know | 1.01 |
| Why do you have this opinion that this person breastfed or is only breastfeeding (without giving water, nor other liquids, milk, or small bites of food) for the first 6 months of the baby’s life? |  |
| Because it's good, protective and nutritious for the baby | 68.69 |
| Because she's a good mother | 8.08 |
| She left the baby un protected (regarding health) | 1.01 |
| She had lots of experience | 4.04 |
| She had time | 1.01 |
| She did it because of lack of money | 0 |
| She did wrong because baby needs other foods | 0 |
| Breast milk alone doesn't feed the baby | 4.04 |
| That you have to give water | 0 |
| That you have to give tea infusion | 0 |
| That she is spoiling the baby | 0 |
| It isn't good for/weakens the mother | 1.01 |
| Doctor doesn't recommend it | 0 |
| Other | 9.09 |
| Don't know | 1.01 |
| Of the above, what do you consider most important that this person breastfed or is only breastfeeding (without giving water, nor other liquids, milk, or small bites of food) for the first 6 months of the baby’s life? |  |
| Because it's good, protective and nutritious for the baby | 62.89 |
| Because she's a good mother | 11.34 |
| She left the baby un protected (regarding health) | 2.06 |
| She had lots of experience | 3.09 |
| She had time | 2.06 |
| She did it because of lack of money | 1.03 |
| She did wrong because baby needs other foods | 4.12 |
| Breast milk alone doesn't feed the baby | 1.03 |
| That you have to give water | 0 |
| That you have to give tea infusion | 0 |
| That she is spoiling the baby | 0 |
| It isn't good for/weakens the mother | 1.01 |
| Doctor doesn't recommend it | 0 |
| Other | 10.31 |
| Don't know | 1.01 |
| If you have any questions about how to feed your baby only with breast milk (without giving water, nor other liquids, milk, or small bites of food), where do you look for advice/help? |  |
| Books or magazines | 1.25 |
| Doctor | 63.75 |
| Nurse | 16.56 |
| Health promotor | 3.44 |
| Oportunidades [Prospera] Vocal | 0.62 |
| Friends or family | 10.94 |
| Mother | 42.19 |
| Mother-in-law | 20.31 |
| Partner/spouse | 1.25 |
| Television/radio | 0.31 |
| Internet | 5.94 |
| Specialist (Pediatritian) | 5.31 |
| Other | 7.81 |
| Of the above options, which is the most important? |  |
| Books or magazines | 9.43 |
| Doctor | 47.14 |
| Nurse | 4.71 |
| Health promotor | 1.01 |
| Oportunidades [Prospera] Vocal | 0.34 |
| Friends or family | 2.36 |
| Mother | 19.19 |
| Mother-in-law | 4.71 |
| Partner/spouse | 0.67 |
| Television/radio | 0 |
| Internet | 1.68 |
| Specialist (pediatritian) | 3.7 |
| Other | 5.05 |
| Some women prefer to not feed their baby only with breast milk. In your opinion, how are the women (in attitude or they way they are) that don’t want to only give breast milk to their baby for the first 6 months? |  |
| They're very young | 2.8 |
| It’s her first baby | 4.36 |
| They're lazy | 7.79 |
| They work | 16.2 |
| They don't take care of their baby | 20.56 |
| They're good mothers | 0 |
| They're worried about their baby | 0.93 |
| Other | 41.74 |
| Don't know | 5.61 |
| What of the above opinions would be the reasons most important for you that women don’t give only breast milk to their baby? |  |
| They're very young | 1.57 |
| It’s her first baby | 4.7 |
| They're lazy | 7.21 |
| They work | 16.3 |
| They don't take care of their baby | 19.75 |
| They're good mothers |  |
| They're worried about their baby | 0.94 |
| Other | 44.2 |
| Don't know | 5.33 |
| Could anyone change your opinion of or make you change the way you feed your baby? |  |
| Yes | 35.83 |
| No | 63.24 |
| Who could advise you that would make you change the way you feed your baby? |  |
| No one (myself) |  |
| Spouse/partner | 2.61 |
| Mother | 19.13 |
| Mother-in-law | 5.22 |
| Friend/neighbor | 0 |
| Oportunidades [Prospera] Vocal | 0 |
| Health Auxiliary | 0 |
| Doctor | 52.17 |
| Nurse | 3.48 |
| Pediatrician/specialist/nutritionist | 13.04 |
| Other | 4.35 |
| Don't know | 0 |
| How important is it to you what your spouse/partner thinks of giving only breast milk (without water nor any other liquid, milk, or small bites of food) for the first 6 months? |  |
| Very important | 3.65 |
| Important | 5.98 |
| Not very important | 38.21 |
| Not important at all | 45.85 |
| Don't know | 6.31 |
|  |  |
| How important is it to you what your mother thinks of giving only breast milk (without water nor any other liquid, milk, or small bites of food) for the first 6 months? |  |
| Very important | 5 |
| Important | 10.31 |
| Not very important | 39.38 |
| Not important at all | 41.56 |
| Don't know | 3.75 |
| How important is it to you what your mother-in-law thinks of giving only breast milk (without water nor any other liquid, milk, or small bites of food) for the first 6 months? |  |
| Very important | 11.04 |
| Important | 13.96 |
| Not very important | 36.36 |
| Not important at all | 23.7 |
| Don't know | 14.94 |
| How important is it to you what your doctor thinks of giving only breast milk (without water nor any other liquid, milk, or small bites of food) for the first 6 months? |  |
| Very important | 1.25 |
| Important | 2.18 |
| Not very important | 28.35 |
| Not important at all | 67.91 |
| Don't know | 0.31 |
| How important is it to you what your nurse thinks of giving only breast milk (without water nor any other liquid, milk, or small bites of food) for the first 6 months? |  |
| Very important | 2.8 |
| Important | 11.21 |
| Not very important | 44.55 |
| Not important at all | 41.12 |
| Don't know | 0.31 |
| Who do you ask for advice or listen to most about feeding your infant? |  |
| Husband | 4.69 |
| Mother | 23.75 |
| Mother-in-law | 7.81 |
| Sister/aunt | 2.81 |
| Doctor | 50 |
| Nurse | 2.19 |
| other | 8.75 |
| What do you think your family member would say about giving only breast milk (without water nor any other liquid, milk, or small bites of food) for the first 6 months? |  |
| Agree | 60.5 |
| Not Agree | 35.11 |
| Don't know | 4.39 |
| Why is your family member not in agreement with giving only breast milk (without water nor any other liquid, milk, or small bites of food) for the first 6 months? |  |
| Because the baby would still be hungry | 34.92 |
| Because it's not taking good care of the baby | 2.38 |
| Because you should give other milk in addition | 3.17 |
| Because you should also give water/tea infusion | 29.37 |
| Because the baby should eat other foods | 11.9 |
| Because it weakens the mother |  |
| Because the mother has to work | 0.79 |
| No opinion |  |
| They wouldn't disagree | 2.38 |
| Other | 5.56 |
| Don't know | 9.52 |
|  |  |
