# Supplementary material for: Breastfeeding practices, beliefs, and social norms in low-resource communities in Mexico: Insights for how to improve future promotion strategies
Source: PLoS One. 2017 Jul 3;12(7):e0180185. doi: 10.1371/journal.pone.0180185 (PMC5495390; doi:10.1371/journal.pone.0180185)
Supplement: S1 Appendix — (PDF) [file pone.0180185.s005.pdf]

**Instituto Nacional de Salud Pública**
**Estrategia Integral de Atención a la Nutrición –Oportunidades 2013**
**Ampliación propuesta de comunicación**

| **1.0 IDENTIFICADOR DEL ENTREVISTADO**<br>**2.0 IDENTIFICADOR DEL CUESTIONARIO** |
|---|
| Folio de la persona encuestada: ……………………………………\|___\|___\|___\|___\|___\|<br><br>Folio de Oportunidades: \|___\|___\|___\|___\|___\|___\|___\|___\|___\|___\|___\|___\|___\|___\|___\|___\|<br><br>Número progresivo del cuestionario:……………………………\|___\|___\|___\|___\| |

| **3.0 IDENTIFICADOR DE LA LOCALIDAD** | |
|---|---|
| NOMBRE | ___________________________ URBANA( 1 ) RURAL( 2 ) |
| LOCALIDAD | \|___\|___\|___\|___\| |
| MUNICIPIO | \|___\|___\|___\| |
| ENTIDAD FEDERATIVA | \|___\|___\| |

| **4.0 RESULTADO DE LA ENCUESTA** | | | |
|---|---|---|---|
| **4.1** Nombre y clave del encuestador | ______________________<br>\|___\|___\| | **4.2** Fecha (anote dd y mm) | \|___\|___\|-\|___\|___\|-\|_1_\|_3_\| |
| **4.3** Hora de inicio | \|___\|___\| : \|___\|___\| | **4.4** Hora de término | \|___\|___\| : \|___\|___\| |
| **4.5.1**<br>1-Embarazada<br>2-Madre <6m \|____\|<br>3-Madre <12<br>4-Madre 12 a 24m | **4.5.2** Resultado de encuesta (*)<br>\|____\| | 1. Encuesta completa (*)<br>2. Incompleta. Se negó a continuar con la encuesta.<br>3. Informante inadecuado.<br>4. Entrevista aplazada a petición del informante (se concluirá en otro momento).<br>5. Se negó a dar información.<br>6. Informante adecuado no disponible (no se hará encuesta).<br>7. Otro (esp)___________________________ | |
| **¿Cuál es su nombre?** (ANOTE NOMBRE DE LA MUJER)<br>_____________________________________<br>APELLIDO PATERNO APELLIDO MATERNO<br>______________________<br>NOMBRE(S) | | **4.6** Usted ¿es beneficiaria de Oportunidades?<br>Si=1 \|___\|<br>No=2 | **4.7** ¿Qué edad tiene?<br>\|___\|___\|<br>No sabe/ no responde=99 |
| **EMBARAZO ACTUAL**<br>**4.8** Usted ¿está actualmente embarazada?<br>Si=1 \|___\|<br>No=2 } **Pase a 4.10**<br>No sabe=9 } | | **4.9** ¿Es su primer embarazo?<br>Si=1 → **Pase a Secc. 5** \|___\|<br>No=2 | |
| **NIÑO <24 MESES BENEFICIARIO DE OPORTUNIDADES**<br>**4.10** ¿Cuántos hijos(as) (vivos) tiene Usted? \|___\|<br>9=No sabe/ no responde | | **4.11** ¿Qué edad tienen su hijo(a) menor de 2 años? \|___\| años \|___\|___\|meses<br>Edad de **NOMBRE [<1 año (2 años) beneficiario de Oportunidades**]<br>*[Si tiene menos de 1 año, anotar 0 en años y anotar número de meses; si tiene menos de un mes poner 00 en años y 00 en meses]* | |
| **4.12** ¿Cuál es el sexo de su hijo(a)? (MARQUE CON UNA CRUZ)<br>Masculino ( 1 ) Femenino ( 2 ) | | **¿Cuál es el NOMBRE de su hijo menor de 2 años (beneficiario de *Oportunidades*)?**<br>[ANOTE NOMBRE DEL NIÑO(A) MENOR DE 2 AÑOS (BENEFICIARIO DE *OPORTUNIDADES*)]<br>_____________________________________________<br>APELLIDO PATERNO APELLIDO MATERNO NOMBRE(S) | |
| **4.13 ¿Qué lugar ocupa (NOMBRE) entre sus hermanos menores de 5 años?** | | | \|____\|<br>9= No sabe/ no responde |
| **[ENCUESTADOR: SI LA RESPUESTA FUE AFIRMATIVA PARA LA PREGUNTAS 4.8; EDAD≥6 ó ≥12 meses EN 4.10 PROCEDA A APLICAR LA ENCUESTA]** | | | |

**[APLIQUE A MUJERES USUARIAS DE SERVICIOS DE SALUD BENEFICIARIAS DEL PROGRAMA OPORTUNIDADES –MADRES DE NIÑOS MENORES DE 1 AÑOS Y EN CONSULTA PRENATAL, DE PREFERENCIA PRIMERIZAS JÓVENES <20 AÑOS]**

| | |
|---|---|
| **5. CARACTERISTICAS GENERALES (socio-demográficas) (APLICAR A TODAS LAS MUJERES)**<br>**“Primero quiero comentar con Usted sobre algunas cosas generales de su familia y hogar”.** | |
| 5.1 ¿Cuál es su estado civil? | Es soltera= 1<br>Es casada= 2<br>Vive en unión libre= 3 \|___\|<br>Es viuda= 4<br>Es separada = 5<br>Es divorciada= 6 |
| 5.2 ¿Cuál fue su último grado o año de estudios?<br><br>[Indique máximo grado de estudios concluido] | Ninguna=1<br>Kinder o preescolar=2<br>Primaria=3<br>Secundaria=4 \|___\|<br>Carrera Técnica=5<br>Preparatoria=6<br>Universidad=7<br>Otro=8 (especificar) ________________________________<br>No sabe=9 |
| 5.3 ¿Usted habla alguna lengua indígena? | Si=1<br>No=2 \|___\|<br>No sabe=9 |
| 5.4 ¿Con quién vive Ud. en su casa?<br><br>**[Utilice código 1=Si; 2=No; 9=No sabe]**<br><br>*[Todas las casillas deberán tener dato]* | 1. Esposo(a) o pareja..........................( )<br>2. Hijo(a)(s) …………………….......…( )<br>3. Padre o madre …………………......( )<br>4. Abuelo ......…………………….........( )<br>5. Abuela....…………………………......( )<br>6. Hermano(s) ……......………......……( )<br>7. Hermana(s).....…………………..…..( )<br>8. Cuñado(a) ……………………..……( )<br>9. Yerno o nuera ……………… ……… ( )<br>10. Nieto(a) …………………………..…( )<br>11. Sobrino(a) ………………………......( )<br>12. Primo(a) ………..…….……………. ( )<br>13. Suegro ……………………….... ….( )<br>14. Suegra………………………………..( )<br>14. Tío(a) ………………………………. ..( )<br>15. Hijo(a) adoptivo ……………………..( )<br>16. Padrastro o madrastra ……........…..( )<br>17. Hijastro(a) o entenado(a) ............. ( )<br>18. Abuelo(a) político(a) ………...……..( )<br>88. Otro parentesco (especificar) ..... ( )______________________ |
| 5.5 ¿Tiene radio en su casa? | Si=1<br>No=2 \|____\|<br>No sabe=9 |
| 5.6 Usted ¿escucha alguna vez la radio? | Si=1<br>No=2 } **Pase a 5.10** \|____\|<br>No sabe=9 } |
| 5.7 ¿Cuántos días a la semana escucha la radio? | \|____\| días por semana<br>8= Otro (especificar) _____________________________<br>9= No sabe /No recuerda /No responde |
| 5.8 ¿Qué clase de programa de radio escucha Usted **más seguido**?<br><br>[ANOTE LAS DOS MÁS IMPORTANTES] | 1= Noticias<br>2= Música<br>3= Infantil \|____\|<br>4= Religioso<br>5= Deportes \|____\|<br>6= Radionovela<br>7= Temas de salud o enfermedad<br>8= Otro (especificar)____________________________<br>9= No sabe /No recuerda /No responde |

| | |
|---|---|
| 5.9 Generalmente, ¿A qué hora escucha radio?<br><br>**[Utilice código 1=Si; 2=No; 9=No sabe]**<br><br>*[Todas las casillas deberán tener dato]* | 1= 0:00 - 5:59 hrs………………………..( )<br>2= 6:00 - 8:59 hrs………………………..( )<br>3= 9:00 - 11:59 hrs……………………..( )<br>4= 12:00 - 14:59 hrs……………………..( )<br>5= 15:00 - 17:59 hrs……………………..( )<br>6= 18:00 - 23:59 hrs……………………..( )<br>8= Otro (especificar)___________________________...( ) |
| 5.10 Usted ¿tiene televisión en su casa? | Si=1<br>No=2 \|____\|<br>No sabe=9 |
| 5.11 Usted ¿Ve alguna vez la televisión? | Si=1<br>No=2 } **Pase a 5.16** \|____\|<br>No sabe=9 |
| 5.12 ¿Cuántos días a la semana ve televisión? | \|____\| días por semana<br><br>8= Otro (especificar) ____________________________<br>9= No sabe /No recuerda /No responde |
| 5.13 ¿Qué canales de televisión ve Usted con más frecuencia?<br><br>[ANOTE LAS DOS MÁS IMPORTANTES, **EL CÓDIGO** SE ASIGNARÁ EN CAPTURA] | 1. ________________________________________ \|____\|<br>2. ________________________________________ \|____\|<br><br>8= No aplica/No procede/Nunca ve televisión<br>9= No sabe /No recuerda /No responde |
| 5.14 ¿Qué clase de programa ve Usted **más seguido** en la televisión?<br><br>[ANOTE LAS DOS MÁS IMPORTANTES] | 1= Noticias<br>2= Música<br>3= Infantil \|____\|<br>4= Religioso<br>5= Deportes \|____\|<br>6= Telenovela<br>7= Temas de salud o enfermedad<br>8= Otro (especificar)____________________________<br>9= No sabe /No recuerda /No responde |
| 5.15 Generalmente ¿A qué hora ve televisión?<br><br>**[Utilice código 1=Si; 2=No; 9=No sabe]**<br><br>*[Todas las casillas deberán tener dato]* | 1= 0:00 - 5:59 hrs………………………..( )<br>2= 6:00 - 8:59 hrs………………………..( )<br>3= 9:00 - 11:59 hrs……………………..( )<br>4= 12:00 - 14:59 hrs……………………..( )<br>5= 15:00 - 17:59 hrs……………………..( )<br>6= 18:00 - 23:59 hrs……………………..( )<br>8= Otro (especificar)___________________________...( ) |
| 5.16 Usted ¿tiene computadora en su casa? | Si=1<br>No=2 \|___\|<br>No sabe=9 |
| 5.17 Usted ¿Alguna vez usa una computadora con internet (donde sea)? | Si=1<br>No=2 } **Pase a 5.21** \|___\|<br>No sabe=9 |
| 5.18 ¿Cuántos días a la semana usa computadora con internet? | \|____\| días por semana<br><br>8= Otro (especificar) ____________________________<br>9= No sabe /No recuerda /No responde |
| 5.19 ¿Qué páginas de internet usa Usted con más frecuencia? [ANOTE LAS DOS MÁS IMPORTANTES, EL **CÓDIGO** SE ASIGNARÁ EN CAPTURA] | 1. ______________________________________ \|____\|<br>2. ______________________________________ \|____\|<br>8= No aplica/No procede/Nunca usa internet<br>9= No sabe /No recuerda /No responde |

| | |
|---|---|
| 5.20 ¿Qué clase de información busca Usted más seguido en internet?<br><br>[ANOTE LAS DOS MÁS IMPORTANTES] | 1= Noticias<br>2= Entretenimiento (deporte, música) \|____\|<br>3= Correo electrónico<br>4= Redes sociales \|____\|<br>5= Temas de salud o enfermedad<br>8= Otro (especificar) ___________________________<br>9= No sabe /No recuerda /No responde |
| 5.21 ¿Usted tiene teléfono celular (personal o en su casa)? | Si=1<br>No=2 \|____\|<br>No sabe=9 |
| 5.22 ¿Alguna vez usa Usted el teléfono celular? | Si=1<br>No=2 } **Pase a 5.25** \|____\|<br>No sabe=9 |
| 5.23 ¿Cada cuánto usa el teléfono celular? | \|____\| días por semana<br><br>8= Otro (especificar) ___________________________<br>9= No sabe /No recuerda /No responde |
| 5.24 Usted ¿qué clase de publicidad o anuncios recibe más seguido en el teléfono celular?<br><br>[ANOTE LAS DOS MÁS IMPORTANTES] | 1= Noticias<br>2= Entretenimiento (música, deportes, etc.) \|____\|<br>3= Temas de salud o enfermedad<br>4= Mensajes de su compañía telefónica \|____\|<br>8= Otro (especificar) ___________________________<br>9= No sabe /No recuerda /No responde |
| 5.25 ¿Ha visto o escuchado algún mensaje relacionado con **dar pecho** al bebé o a su bebé? | Si=1<br>No=2 } **Pase a 5.28** \|____\|<br>No sabe/no recuerda=9 |
| 5.26 ¿Dónde fue la última vez que **vio o escuchó** un mensaje relacionado con **dar pecho** al bebé?<br><br>*[No lea, ni sugiera, registre la que indique el encuestado]*<br><br>**Encuestador: En caso de que NO REFIERA la opción 06, 07 u 08, PASAR A PREG. 5.27.** | Televisión…………………………………………01<br>Radio…………………….……………………02<br>Periódico/revista……………………………03 \|____\|____\|<br>Internet……………………………………….04<br>Calle o avenida (cartel, anuncio)............05<br>Hospital …………………………………….06<br>Clínica privada…………………….………..07<br>Centro de Salud…………………….………..08<br>Otro…88 (especificar)______________________________<br>No sabe……………………………………….99 |
| *5.26a* En caso que haya referido la opción 06, 07 u 08 en la pregunta anterior, especificar quién se lo dio.<br>*(El código se asignará en la captura)* | ¿Quién se lo dió?_____________________________ \|____\| |
| 5.27 ¿Cuándo fue la última vez que **vio o escuchó** un mensaje relacionado con **dar pecho** al bebé en televisión, radio o periódicos?<br><br>*[No lea, ni sugiera, registre la que indique el encuestado]* | En los últimos 7 días…………………………………1<br>Hace más de 7 días, pero menos de 1 mes….2<br>Entre 1 y 3 meses………………………………………3<br>Entre 3 y 6 meses………………………………………4 \|____\|<br>Hace más de 6 meses…………………………………5<br>Nunca…………………………………………………………6<br>No sabe………………………………………………………9 |
| 5.28 ¿Ha visto o escuchado algún mensaje relacionado con **leche de fórmula o leche de bote (tipo Nido)** para alimentar al bebé? | Si=1<br>No=2 } **Pase a Secc. 6** \|____\|<br>No sabe/no recuerda=9 |

| | |
|---|---|
| 5.29 ¿Dónde fue la última vez que **vio o escuchó** un mensaje relacionado con leche de fórmula o leche de bote **(tipo Nido)** de leche para alimentar al bebé?<br><br>*[No lea, ni sugiera, registre la que indique el encuestado]*<br><br>**Encuestador: En caso de que NO REFIERA la opción 06, 07 u 08, PASAR A PREG. 5.30.** | Televisión…………………………………………..01<br>Radio…………………………………………………..02<br>Periódico/revista………………………………..03 \|____\|____\|<br>Internet……………………………………………..04<br>Calle o avenida (cartel, anuncio)...........05<br>Hospital ……………………………………………..06<br>Clínica privada………………………………......07<br>Centro de Salud……………………………..……08<br>Otro… 88 (especificar)______________________________<br>No sabe…………………………………………….99 |
| *5.29a* En caso que haya referido la opción 06, 07 u 08 en la pregunta anterior, especificar quién se lo dio.<br>*(El código se asignará en la captura)* | ¿Quién se lo dio?______________________________ \|____\| |
| 5.30 ¿Cuándo fue la última vez que **vio o escuchó** un mensaje relacionado con leche de fórmula o bote para alimentar al bebé en televisión, radio o periódicos?<br><br>*[No lea, ni sugiera, registre la que indique el encuestado]* | En los últimos 7 días…………………………………..1<br>Hace más de 7 días, pero menos de 1 mes….2<br>Entre 1 y 3 meses……………………………………..3<br>Entre 3 y 6 meses……………………………………..4 \|____\|<br>Hace más de 6 meses………………………………..5<br>Nunca…………………………………………………………6<br>No sabe……………………………………………………..9 |

**6.SECCIÓN INFORMACIÓN PRENATAL (APLICAR A TODAS LAS MUJERES)**
**"Ahora me gustaría hablar con Usted sobre dónde lleva o llevó el control de su embarazo de [NOMBRE]".**

| | |
|---|---|
| 6.1 ¿Dónde realiza (realizó) el control de su embarazo?<br><br>*[No lea, ni sugiera, registre la que indique el encuestado]* | Centro de salud de la SSA……………..1<br>Clínica del IMSS *Oportunidades*……..2<br>Hospital de la SSA………………….……..3 \|___\|<br>Partera……………………………………..….4<br>Consultorio o clínica privada…….…..5<br>Otro=8 (especificar)__________________________________<br>No sabe………………………………………….9 |
| 6.2 Durante su embarazo ¿Alguien le habló (o le han hablado) sobre dar pecho a su bebé mientras estaba embarazada? | Si=1<br>No=2 } ***Pase a 6.5*** \|___\|<br>No sabe=9 |
| 6.3 ¿En dónde le hablaron sobre dar pecho a su bebé?<br><br>*[No lea, ni sugiera, registre la que indique el encuestado]* | Centro de Salud de SSA.………………1<br>Clínica del IMSS…………………………..2<br>Hospital de la SSA……………………….3<br>Partera……………………………………….4 \|___\|<br>Talleres de Oportunidades………….5<br>Talleres del Seguro Popular ("yo puedo, yo quiero")…….6<br>Consultorio o Clínica Privada……….7<br>Otro =8 (especificar)__________________________________<br>No sabe……………………………………….9 |
| 6.4 Durante su embarazo ¿Quién le habló sobre dar pecho a su bebé?<br><br>*[Registre la más importante. No lea, ni sugiera, registre la que indique el encuestado]* | Médico………………………………………01<br>Enfermera…………………………….……02<br>Promotor de Salud……………………..03<br>Auxiliar en Salud………………………..04 \|___\|___\|<br>Vocal de Oportunidades…………….05<br>Partera……………………………….……06<br>Madre…………………………………..…07<br>Suegra…………………………………..…08<br>Familiar (hermana, tía, etc.)……….09<br>Otro=88 (especificar)__________________________________<br>No sabe…………………………………….99 |

| | |
|---|---|
| 6.5 Durante su embarazo ¿Alguien le recomendó dar leche de fórmula (o bote) a su bebé? | Si=1<br>No=2 } ***Pase a 6.8*** \|___\|<br>No sabe=9 |
| 6.6 Durante su embarazo ¿En dónde le hablaron sobre alimentar con leche de fórmula a su bebé?<br><br>*[No lea, ni sugiera, registre la que indique el encuestado]* | Centro de Salud de SSA.....................1<br>Clínica del IMSS *Oportunidades*.......2<br>Hospital de la SSA.............................3<br>Partera...............................................4 \|___\|<br>Talleres de Oportunidades..............5<br>Talleres del Seguro Popular ("yo puedo, yo quiero").......6<br>Consultorio o Clínica Privada...........7<br>Otro=8 (especificar)___________________________________<br>No sabe.............................................9 |
| 6.7 Durante su embarazo ¿Quién le habló sobre dar leche de fórmula a su bebé?<br><br>*[No lea, ni sugiera, registre la que indique el encuestado]* | Médico.............................................01<br>Enfermera.......................................02<br>Promotor de salud.........................03<br>Auxiliar en salud.............................04 \|___ \|___\|<br>Vocal de Oportunidades.................05<br>Partera.............................................06<br>Madre..............................................07<br>Suegra.............................................08<br>Familiar (hermana, tía, etc.)..........09<br>Otro=88 (especificar)___________________________________<br>No sabe...........................................99 |
| 6.8 Para Usted, ¿Qué es dar de comer sólo pecho a su bebé?<br><br>(Utilice sinónimos, como: *puro, solamente, únicamente, exclusivamente* dar pecho) | Dar sólo pecho, no incluir agua ni otros líquidos u otra leche=1<br>Dar sólo pecho, no otra leche=2<br>Dar pecho más agua o té=3 \|___\|<br>Dar pecho y fórmula=4<br>Dar pecho y ocasionalmente probaditas de alimentos=5<br>Otro (especificar)=8____________________________________<br>No sabe=9 |
| 6.9 ¿Durante el embarazo Usted planea(ó) dar sólo pecho a su bebé sin darle líquidos, ni otra leche, ni probaditas de alimentos?<br><br>*[INDAGAR SOBRE LACTANCIA MATERNA EXCLUSIVA,* ***sólo*** *dar pecho al bebé]* | Si=1<br>No=2 } **Pase a 6.11** \|____\|<br>No sabe=9 |
| 6.10 ¿Por cuánto tiempo planea(ó) dar sólo pecho a su bebé, sin darle líquidos, ni otra leche, ni probaditas de alimentos? | \|___\|____\|meses<br>No responde o no especifica = 99 |
| 6.11 ¿Por cuánto tiempo planea (o planeó) dar pecho a su bebé junto con otros alimentos? | \|___\|____\|meses<br>No responde o no especifica = 99 |
| 6.12 Durante su embarazo ¿Ud. planea (o planeó) dar fórmula u otra leche (diferente a la materna) a su bebé? | Si=1<br>No=2 } **Pase a 6.16** \|____\|<br>No sabe=9 |
| 6.13 Durante su embarazo ¿Cuál fórmula o leche planea(ó) dar a su bebé? | Liconsa=1<br>Nido=2 \|____\|<br>Nan, Enfamil, Enfagrow, Nursoy, etc. =3<br>Leche Lala, Alpura, etc.=4<br>Otro=8 (esp)__________________________<br>No sabe/ no recuerda=9 |
| 6.14 Durante su embarazo ¿Hasta a qué edad planea(ó) dar fórmula a su bebé? | \|___\|____\|meses<br><br>No responde o no especifica = 99 |

| | |
|---|---|
| 6.15 ¿Por qué planea(ó) dar fórmula u otra leche (diferente a la materna) a su bebé?<br><br>*[Registre la más importante. No lea, ni sugiera, registre la que indique el encuestado]* | Porque trabaja……………………………….1 \|____\|<br>Porque es lo mejor para el bebé……..2<br>Porque alimenta al bebe…………………..3<br>Porque sólo el pecho no lo llena……..4<br>Porque llena …………………………………..5<br>Porque es fácil de preparar………………6<br>Porque lo recomendó el médico/enfermera………..7<br>Otro (especificar)_________________________....8<br>No sabe………………………………………………………..9 |
| **Ahora me gustaría hablar sobre algunos aspectos de salud que presenta (o presentó) durante su embarazo (con NOMBRE)".** | |
| 6.16 Usted ¿sabe cuánto mide de estatura?<br><br>[ENCUESTADOR REGISTRE EN METROS Y CM.] | Si=1<br>No=2 \|___\|<br>No sabe=9<br><br>Si contesto **código 1**, pregunte y registre: ¿Cuánto?<br>Talla \|____\|.\|____\|____\|mts<br>No recuerda = 9.99 |
| 6.17 Usted ¿sabe cuál fue su peso antes o al inicio del embarazo (primer trimestre)? | Si=1<br>No=2 \|___\|<br>No sabe=9<br><br>Si contesto **código 1**, pregunte y registre: ¿Cuánto?<br>Peso \|____\|____\|____\|.\|____\|____\|kgs<br>No recuerda = 999.99 |
| 6.18 ¿Le dijeron cuánto subió o cuántos kilos ganó (o ha ganado) durante el embarazo? | Si=1<br>No=2 } \|___\|<br>No sabe=9 } **Pase a siguiente Secc.**<br><br>Peso ganado\|___\|\|___\|.\|___\|\|___\|kgs<br>**(Anotar el peso que comenta la señora que ganó en su último embarazo o que ha ganado en el embarazo actual).**<br>No sabe o no recuerda = 99.99] |
| **7. Sección Lactancia Materna Exclusiva (LME)**<br>**[APLIQUE A TODAS LAS MUJERES ENCUESTADAS] NOMBRE=___________________________________**<br>**"Ahora me gustaría hablar sobre la alimentación de su bebé o de [NOMBRE] en los primeros meses de vida".** | |
| 7.1 ¿Usted sabe o le han dicho por cuánto tiempo se recomienda dar sólo pecho después que nace el bebé, sin agua, té u otros líquidos? | Si=1<br>No=2 } \|___\|<br>No sabe/no recuerda=9 } **Pase a 7.3** |
| 7.2 ¿Cuánto tiempo después que nace el bebé le han dicho o ha escuchado que se debe dar sólo pecho al bebé (sin agua, te u otros líquidos)? | \|____\|____\| meses<br><br>No sabe, no recuerda = 99 |
| 7.3 Usted, ¿Qué opina de darle sólo pecho a su bebé por **6 meses**? | Está de acuerdo= 1<br>No está de acuerdo=2 →**Pase a 7.6** \|___\|<br>No sabe=9 →**Pase a 7.8** |
| 7.4 ¿Por qué está de acuerdo en darle sólo pecho a su bebé por 6 meses (sin agua ni ningún líquido, ni otra leche, ni probaditas de alimentos)?<br><br>*[REGISTRE LOS PRIMEROS TRES QUE MENCIONE]*<br><br>*[Cancele casillas que no utilice]* | Porque es lo mejor para el bebé=01 \|___\|___\|<br>Porque es suficiente y adecuada=02<br>Porque está bien=03 \|___\|___\|<br>Porque le da defensas contra enfermedades=04<br>Porque se le da amor=05 \|___\|___\|<br>Porque es barato y práctico=06<br>Porque lo nutre y ayuda en su crecimiento=07<br>Otro (esp)=08<br>No sabe=99 |
| 7.5 De lo que me acaba de mencionar, ¿Cuál opinión sería la más importante para Usted?<br>*[Si sólo menciona **una** repita el código]* | \|___\|___\|<br><br>**[AL TERMINAR PASE A 7.8]** |

| | |
|---|---|
| 7.6 ¿Por qué está en desacuerdo en darle sólo pecho a su bebé por 6 meses (sin agua ni ningún líquido, ni otra leche, ni probaditas de alimentos)?<br><br>*[REGISTRE LOS PRIMEROS TRES QUE MENCIONE]*<br><br>*[Cancele casillas que no utilice]* | Porque no tengo suficiente leche=01<br>Porque se quedaría con hambre=02 \|___\|___\|<br>Porque le daría sed=03<br>Porque no se alimentaria bien=04<br>Porque no crecería lo suficiente bien=05 \|___\|___\|<br>Porque no está bien=06<br>Porque la madre trabaja=07<br>Porque es cansado dar pecho al bebe=08 \|___\|___\|<br>Otro (especifique)=88________________________<br>No sabe=99 |
| 7.7 De lo que me acaba de mencionar, ¿Cuál opinión sería la más importante para Usted?<br>*[Si sólo menciona **una** repita el código]* | \|___\|___\| |
| 7.8 Para los primeros SEIS meses de vida, ¿Usted planea (o planeó) darle sólo pecho al niño(a) (sin agua ni ningún líquido, ni otra leche, ni probaditas de alimentos)?<br><br>[MUESTRE TARJETA DE OPCIONES DE RESPUESTA] | No planea(ó) dar lactancia materna (LM) = 1<br>Estoy pensando (o pensó) en dar LM = 2<br>Estoy dando (o dió) LM = 3 \|____\|<br>Voy a seguir (o siguió) dando LM = 4<br>Otra respuesta diferente = 5 |
| 7.9 ¿Por cuánto tiempo planea(ó) dar sólo leche materna a su hijo(a) (sin agua ni ningún líquido, ni otra leche, ni probaditas de alimentos)? | \|___\|____\|meses<br><br>99= No sabe/ no responde |
| 7.10 ¿Alguien puede opinar o hacerle cambiar de idea sobre la alimentación del bebé? | Si=1<br>No=2<br>No sabe=9 } **Pase a 7.12** \|____\| |
| 7.11 ¿Quién podría aconsejarla para cambiar la alimentación del bebé?<br><br>[REGISTRE HASTA TRES OPCIONES DE RESPUESTA, en orden de importancia] | Nadie, ella misma=01<br>Esposo/pareja=02 \|___\|___\|<br>Madre=03<br>Suegra=04<br>Amiga/vecina=05 \|___\|___\|<br>Vocal de Oportunidades=06<br>Auxiliar de salud=07<br>Médico=08<br>Enfermera=09 \|___\|___\|<br>Pediatra ,especialista, nutriólogo= 10<br>Otro (especifique)=88___________________________<br>No sabe=99 |
| **[APLIQUE SI LA ENCUESTADA REFIERE TENER PAREJA O ESPOSO, DE LO CONTRARIO PASE A PREG. 7.13]**<br><br>7.12 Para Usted, ¿Qué tan importante es lo que piense su **esposo/pareja** sobre dar sólo pecho (sin agua, ni ningún líquido, ni otra leche, ni probaditas de alimentos) al bebé?<br><br>[MUESTRE TARJETA DE OPCIONES DE RESPUESTA] | Muy importante=4<br>Importante=3 \|____\|<br>Poco importante=2<br>Nada importante=1<br>No sabe=9 |
| 7.13 Para Usted, ¿Qué tan importante es lo que piense **su madre** sobre dar sólo pecho (sin agua ni ningún líquido, ni otra leche, ni probaditas de alimentos)?<br><br>[MUESTRE TARJETA DE OPCIONES DE RESPUESTA] | Muy importante=4<br>Importante=3 \|____\|<br>Poco importante=2<br>Nada importante=1<br>No sabe=9 |
| 7.14 Para Usted, ¿Qué tan importante es lo que piense **su suegra** sobre dar sólo pecho (sin agua ni ningún líquido, ni otra leche, ni probaditas de alimentos)?<br><br>[MUESTRE TARJETA DE OPCIONES DE RESPUESTA] | Muy importante=4<br>Importante=3 \|____\|<br>Poco importante=2<br>Nada importante=1<br>No sabe=9 |

| | |
|---|---|
| 7.15 Para Usted, ¿Qué tan importante es lo que piense **su médico** sobre dar <u>sólo</u> pecho (sin agua ni ningún líquido, ni otra leche, ni probaditas de alimentos)?<br><br>[MUESTRE TARJETA DE OPCIONES DE RESPUESTA] | Muy importante=4<br>Importante=3 \|____\|<br>Poco importante=2<br>Nada importante=1<br>No sabe=9 |
| 7.16 Para Usted, ¿Qué tan importante es lo que piense **su enfermera** sobre dar <u>sólo</u> pecho (sin agua ni ningún líquido, ni otra leche, ni probaditas de alimentos)?<br><br>[MUESTRE TARJETA DE OPCIONES DE RESPUESTA] | Muy importante=4<br>Importante=3 \|____\|<br>Poco importante=2<br>Nada importante=1<br>No sabe=9 |
| 7.17 ¿Usted a quién **le** pregunta o le hace **más** caso en relación a la alimentación de su hijo(a)?<br><br>[Si menciona que a ***NADIE***, **escribirlo en 8**] | Esposo=1<br>Madre=2 \|____\|<br>Suegra=3<br>Hermana/tía=4<br>Médico=5<br>Enfermera=6<br>Otro (esp)=8___________________________<br>No sabe/no responde=9<br><br>**SI NO RESPONDE ALGÚN FAMILIAR, PREGUNTE: DE LOS FAMILIARES ¿CUAL SERÍA EL MÁS IMPORTANTE? Código \|____\|** |
| **PREGUNTE EN REFERENCIA AL <u>FAMILIAR</u> QUIÉN CONSIDERA MÁS IMPORTANTE EN LA PREGUNTA ANTERIOR.**<br><br>7.18 Usted ¿Qué cree que diría su **familiar** si le diera <u>sólo</u> pecho (sin agua, ni ningún líquido, ni otra leche, ni probaditas de alimentos) a su hijo(a) por **6 meses**? | Está de acuerdo= 1 →**Pase a 7.21** \|___\|<br>No está de acuerdo=2<br>No sabe=9 |
| 7.19 ¿Por qué no está de acuerdo su **familiar** de que se le diera <u>sólo</u> pecho a su hijo(a) por 6 meses?<br><br>*[REGISTRE LOS PRIMEROS TRES QUE MENCIONE en orden de prioridad]*<br><br>*[cancele casilla(s) si no la utiliza]* | Porque se queda con hambre=01<br>Porque no lo cuida bien=02 \|____\|____\|<br>Porque le debe dar también otra leche=03<br>Porque le debe dar también agua o té=04 \|____\|____\|<br>Porque también debe comer otros alimentos=05<br>Porque la madre se debilita=06 \|____\|____\|<br>Porque tiene que trabajar=07<br>Nada, no opina=08<br>No estaría de acuerdo=09<br>Otro=88 (especificar) ______________________________<br>No sabe=99 |
| 7.20 Si dio más de una respuesta: ¿Cuál opinión sería la más importante para su **familiar**?<br><br>*[Si solo menciona **una** repita el código]* | \|____\|____\| |
| 7.21 Usted ¿cree que puede dar (o hubiera podido dar) <u>sólo</u> pecho (sin agua, ni ningún líquido, ni otra leche, ni probaditas de alimentos) a su hijo por **6 meses**? | Si=1 →***Pase a 7.24***<br>No=2 \|____\|<br>No sabe=9 |
| 7.22 ¿Por qué piensa que no puede dar <u>sólo</u> pecho (sin agua, ni ningún líquido, ni otra leche, ni probaditas de alimentos por **6 meses** a su bebé?<br><br>[REGISTRE LOS PRIMEROS TRES QUE MENCIONE] | Porque está baja de peso=01<br>Porque no tiene mucha leche=02 \|____\|____\|<br>Porque trabaja=03<br>Porque no quiere=04 \|____\|____\|<br>Porque el niño(a) necesita agua=05<br>Porque el niño(a) necesita té para cólicos=06 \|____\|____\|<br>Porque el niño(a) no crece=07<br>Porque el niño(a) pide otros alimentos=08<br>Otro =88 (especificar) ________________________________<br>No sabe=99 |

<table>
<tr><td>7.23 De las que mencionó, ¿Cuál considera que es la razón más importante por la que Usted no puede dar <u>sólo</u> pecho a su bebé por 6 meses?<br><br>[Si sólo menciona una repita el código]</td><td>|____|____|</td></tr>
<tr><td>7.24 ¿Cuáles cree Usted que son los beneficios/ventajas para el bebé de recibir <u>sólo</u> pecho (sin agua, ni ningún líquido, ni otra leche, ni probaditas de alimentos) por 6 meses?<br><br>[REGISTRE LOS PRIMEROS TRES QUE MENCIONE]</td><td>Se le da amor=01<br>Se le da defensas contra enfermedades=02 |____|____|<br>Es barato y práctico=03<br>Disponible en todos lados=04 |____|____|<br>Es la primera vacuna=05<br>Para que estén sanos=06 |____|____|<br>Para que crezca=07<br>Da nutrientes=08<br>Para prevenir enfermedades crónicas en el futuro=09<br>Es normal/ es costumbre=10<br>Otro=88 (especificar) ________________________<br>No sabe=99</td></tr>
<tr><td>7.25 Si dio más de una respuesta: ¿Cuál sería el más importante de los beneficios para su bebé?<br><br>[Si sólo menciona una repita el código]</td><td>|____|____|</td></tr>
<tr><td>7.26 ¿Cuáles cree Usted que son los beneficios/ventajas para la madre de dar <u>sólo</u> pecho (sin agua, ni ningún líquido, ni otra leche, ni probaditas de alimentos) al bebé por 6 meses?<br><br>[REGISTRE LOS PRIMEROS TRES QUE MENCIONE]</td><td>Es gratis (economía)=01<br>Es práctico (disponible en todos lados)=02 |___|___|<br>La madre baja de peso=03<br>Ayuda a no quedar embarazada (anticonceptivo)=04 |___|___|<br>Disminuye el sangrado posparto=05<br>Disminuye depresión post parto=06 |___|___|<br>Disminuye cáncer de mama=07<br>Disminuye cáncer de útero=08<br>Ninguna/ es normal/ es costumbre=09<br>Otro=88 (especificar) ______________________<br>No sabe=99</td></tr>
<tr><td>7.27 Si dio más de una respuesta ¿Cuál sería el más importante de los beneficios para la madre?<br><br>[Si sólo menciona una repita el código]</td><td>|____|____|</td></tr>
<tr><td>7.28 ¿Qué cree que necesitaría Usted para dar (o haber dado) <u>sólo</u> pecho (sin agua, ningún líquido, ni otra leche, ni probaditas de alimentos) por los primeros 6 meses?<br><br>[REGISTRE LOS PRIMEROS TRES QUE MENCIONE]</td><td>Tiempo=01<br>Que alguien le ayudara a cuidar a |___|___|<br>sus otros hijos =02<br>Que alguien la ayudara en los quehaceres del hogar=03<br>Que alguien le diga cómo hacerlo=04 |___|___|<br>Una buena alimentación (que incluya frutas<br>y verduras)=05 |___|___|<br>Tomar más agua=06<br>Que alguien le explique cómo sacarse la leche=07<br>Nada=08<br>Que el bebé agarre o acepte el pecho=09<br>Otro=88 (especificar) __________________________<br>No sabe=99</td></tr>
<tr><td>7.29 Si dio más de una respuesta, ¿Cuál opinión sería la más importante para dar sólo pecho a su bebé por 6 meses?<br><br>[Si sólo menciona una repita el código]</td><td>|____|____|</td></tr>
</table>

| | |
|---|---|
| 7.30 **Leer:** Algunas mujeres tienen problemas para alimentar a sus bebés con sólo leche materna (sin agua, ni ningún líquido, ni otra leche, ni probaditas de alimentos).<br><br>En su opinión, ¿cuáles considera Usted que son las principales dificultades para dar sólo pecho (sin agua ni ningún líquido, ni otra leche, ni probaditas de alimentos) a los bebés durante sus **6 primeros meses de vida**?<br><br>[REGISTRE LOS PRIMEROS TRES QUE MENCIONE] | Nada o ninguno =01<br>Que el bebé se queda con hambre, la leche no lo llena=02<br>No tiene suficiente leche=03<br>Leche no lo alimenta (no nutritiva)=04 \|____\|____\|<br>Les duelen los pechos=05<br>La falta de tiempo=06<br>La falta de experiencia o practica=07 \|____\|____\|<br>No quiere o decide no dar desde el inicio=08<br>Tienen que ir a trabajar=09<br>Teme se maltraten o deformen los senos=10 \| ____\|____\|<br>No tienen bien formado el pezón=11<br>Otro=88 (especificar)______________________________<br>No sabe=99 |
| 7.31 Si dio más de una respuesta ¿Cuál opinión sería la más importante para dar sólo pecho por **6 meses**?<br><br>*[Si sólo menciona **una** repita el código]* | \|____\|____\| |
| 7.32 **Leer:** A algunas mujeres se les va la leche o corta la leche cuando están dando pecho.<br><br>En su opinión, ¿cuáles serían las principales causas o razones por las que se va la leche?<br><br>[REGISTRE LOS PRIMEROS TRES QUE MENCIONE] | Toman anticonceptivos=1<br>Un susto o coraje=2 \|____\|<br>El bebé come demasiado y se acaba=3<br>No está bien nutrida=4 \|____\|<br>No toma suficiente atole o líquidos=5<br>Otro (esp) =8__________________________ \|____\|<br>No sabe=9 |
| 7.33 Si dio más de una respuesta ¿cuál opinión sería la más importante del porque a algunas mujeres se les va la leche?<br><br>*[Si sólo menciona **una** repita el código]* | \|_____\| |
| 7.34 **Leer:** Algunas mujeres prefieren no alimentar a sus bebés con sólo leche materna.<br><br>En su opinión, ¿cómo son (en actitud o forma de ser) las mujeres que no quieren dar sólo pecho a su bebé en los primeros 6 meses de vida?<br><br>[REGISTRE LOS PRIMEROS TRES QUE MENCIONE] | Son muy jóvenes=1<br>Son primerizas=2 \|____\|<br>Son flojas=3<br>Trabajan=4 \|____\|<br>Descuidadas con los hijos=5<br>Son buenas madres =6 \|____\|<br>Se preocupan por sus hijos=7<br>Otro (esp)=8 ____________________________<br>No sabe=9 |
| 7.35 Si dio más de una respuesta ¿cuál opinión sería la más importante de cómo son las mujeres que no dan sólo pecho por 6 meses?<br><br>*[Si sólo menciona **una** repita el código]* | \|____\|____\| |
| 7.36 ¿Qué cree Usted que pueda motivar o hacerles cambiar de opinión a las mujeres para que le den sólo pecho (sin agua, ni ningún líquido, ni otra leche, ni probaditas de alimentos) a su bebé durante los primeros **6 meses de vida**?<br><br>[REGISTRE LOS PRIMEROS TRES QUE MENCIONE] | Que el bebé crecerá más sano=01<br>Que lo pueda hacer bien=02 \|____\|____\|<br>Que alguien le ayude=03<br>Que tenga tiempo para hacerlo=04 \|____\|____\|<br>Que sepan los beneficios=05<br>No gastar o la economía familiar=06 \|____\|____\|<br>Evita obesidad en el niño=07<br>Evita desnutrición=08<br>Se enferma menos=09<br>Más información (ej. no se deforma el cuerpo)=10<br>Otro =88 (especificar)____________________________<br>No sabe=99 |

| | |
|---|---|
| 7.37 Si dio más de una respuesta, ¿cuál sería la motivación más importante para dar <u>sólo</u> pecho (sin agua ni ningún líquido, ni otra leche, ni probaditas de alimentos) por **6 meses**?<br>*[Si sólo menciona **una** repita el código]* | \|____\|____\| |
| 7.38 ¿Conoce a algún familiar/conocida que haya dado (o esté dando) <u>sólo</u> pecho (sin agua, ni ningún líquido, ni otra leche, ni probaditas de alimentos a sus hijos por los primeros **6 meses** de vida (exclusiva)? | Si=1<br>No=2 } ***pase a 7.44*** \|___\|<br>No sabe=9 |
| 7.39 ¿Por qué cree que su familiar/conocida le dio (o le da) <u>sólo</u> pecho (sin agua, ni ningún líquido, ni otra leche, ni probaditas de alimentos a sus hijos(as)) por los primeros **6 meses**?<br><br>[REGISTRE LOS PRIMEROS TRES QUE MENCIONE] | en el hospital o clínica la orientaron=01<br>porque se lo recomendó el médico=02 \|___\|___\|<br>porque se lo recomendó su mamá=03<br>porque se lo recomendó su suegra=04 \|___\|___\|<br>porque era lo mejor para el niño(a)=05<br>porque tenía experiencia con sus otros hijos(a)=06 \|___\|___\|<br>porque no tiene para comprar otras leches=07<br>porque es buena madre =08<br>porque es floja y no quiere preparar otros alimentos =09<br>le dieron tiempo en el trabajo =10<br>porque el niño(a) acepto o agarro bien el pecho=11<br>porque está joven y sana=12<br>porque tiene bastante leche=13<br>tiene pareja/esposo=14<br>Otro=88 (especificar) ______________________<br>No sabe=99 |
| 7.40 .Si dio más de una respuesta, ¿cuál sería la razón más importante por la que su familiar/conocida diera <u>sólo</u> pecho (sin agua ni ningún líquido, ni otra leche, ni probaditas de alimentos) por 6 meses a su hijo(a)?<br>*[Si sólo menciona **una** repita el código]* | \|____\|____\| |
| 7.41 ¿Qué **opina** sobre que su familiar/ conocida dio (o le da) <u>sólo</u> pecho (sin agua ni ningún líquido, ni otra leche, ni probaditas de alimentos) a sus hijos por los primeros **6 meses** de vida? | Que está bien=1<br>Que no está bien=2 \|___\|<br>No sabe=9 |
| 7.42 ¿Por qué tiene esa opinión sobre que su familiar/ conocida diera (o da) <u>sólo</u> pecho (sin agua ni ningún líquido, ni otra leche, ni probaditas de alimentos) por **6 meses** a su(s) hijo(a)(s)?<br><br>[REGISTRE LOS PRIMEROS TRES QUE MENCIONE] | Porque es bueno, protege y nutre al bebé=01<br>Porque es buena madre=02 \|___\|___\|<br>Que lo dejó muy desprotegido (de salud)=03<br>Que tenía mucha experiencia=04 \|___\|___\|<br>Que tenía tiempo=05<br>Que lo hizo por falta de dinero=06 \|___\|___\|<br>Que hizo mal porque necesita otros alimentos=07<br>La leche materna sola no lo alimenta=08<br>Que hay que darle agua=09<br>Que hay que darle té =10<br>Que lo mal acostumbra=11<br>Que no es bueno para la madre, la debilita=12<br>Médico no lo recomienda=13<br>Otro=88 (especificar) ______________________<br>No sabe=99 |
| 7.43 Si dio más de una respuesta ¿cuál sería la opinión más importante del porque su familiar/ conocida dio (o da) <u>sólo</u> pecho (sin agua ni ningún líquido, ni otra leche, ni probaditas de alimentos) por 6 meses a su bebé?<br><br>*[Si sólo menciona **una** repita el código]* | \|____\|____\| |

| | |
|---|---|
| 7.44 Cuando tiene alguna duda sobre cómo alimentar con sólo pecho a su bebé (sin agua ni ningún líquido, ni otra leche, ni probaditas de alimentos), ¿En dónde busca o pregunta por información?<br><br>**[Utilice código 1=Si; 2=No; 9=No sabe]**<br><br>*[Todas las casillas deberán tener dato]* | 01. Libros o revistas………………………………………… \|___\|<br>02. Médico……………………………………………………… \|___\|<br>03. Enfermera ………………………………………………\|___\|<br>04. Promotor de salud…………………………………\|___\|<br>05. Vocal de Oportunidades…………………………\|___\|<br>06. Amigos o familiares………………………………\|___\|<br>07. Madre……………………………………………………… \|___\|<br>08. Suegra……………………………………………………\|___\|<br>09. Pareja/esposo…………………………………………\|___\|<br>10. Televisión / radio…………………………………\|___\|<br>11. Internet…………………………………………………\|___\|<br>12. Especialista (pediatra)…………………………\|___\|<br>88. Otro (especificar)____________________.... \|___\| |
| 7.45 De estas opciones que mencionó, ¿Cuál es la más importante para Usted?<br>*[Si sólo menciona **una** repita el código]* | \|____\| |
| 7.46 Del 0 al 10, ¿Me puede decir que tan confiada o segura está Usted de que la leche materna está alimentando o alimentó bien a su bebé (o NOMBRE) en los primeros **6 meses**?<br>[MUESTRE TARJETA CON ESCALA DE RESPUESTA] | \|____\|____\|<br>No sabe=99 |
| 7.47 Del 0 al 10, ¿Me puede decir que tan confiada o segura está en que la leche materna es mejor que darle leche comercial o fórmula láctea a su bebé en los primeros **6 meses** de vida?<br>[MUESTRE TARJETA CON ESCALA DE RESPUESTA] | \|____\|____\|<br>No sabe=99 |
| 7.48 Del 0 al 10, ¿Me puede decir que tan confiada o segura está en que la leche comercial o fórmula láctea es mejor que darle leche materna a su bebé en los primeros **6 meses** de vida?<br>[MUESTRE TARJETA CON ESCALA DE RESPUESTA] | \|____\|____\|<br>No sabe=99 |
| **[APLIQUE SI REPORTÓ ≥5 EN LA PREGUNTA ANTERIOR]**<br>7.49 ¿En qué marca de leche pensó para responder a la pregunta anterior? | R=_____________________________ \|___\|<br><br>[El código se asignará en captura] |
| 7.50 Del 0 al 10, ¿Me puede decir que tan confiada o segura está en qué la leche materna alimenta a su bebé después del año?<br>[MUESTRE TARJETA CON ESCALA DE RESPUESTA] | \|____\|____\|<br>No sabe=99 |
| 7.51 ¿Ud. cree que hay alguna ventaja para darle leche de formula al bebe durante los primeros **6 meses** de vida? | Si=1 \|____\|<br>No=2 } **Pase a 7.53**<br>No sabe/ no contesta=9 } |
| 7.52 ¿Cuáles ventajas considera que tiene darle leche de fórmula al bebé durante los primeros **6 meses** de vida?<br><br>[REGISTRE HASTA TRES OPCIONES] | Si no tiene tiempo, porque trabaja………,.1 \|____\|<br>Porque es bueno para el bebé……………….2<br>Porque alimenta al bebe………………………..3 \|____\|<br>Porque se complementa con el pecho……4<br>Porque llena …………………………………….....5 \|____\|<br>Es fácil de preparar………………………………6<br>Otro (especificar)__________________________.....8<br>No sabe…………………………………………………………….9 |
| 7.53 Usted sabe o le han dicho ¿Cuánto tiempo en total hay que dar pecho a su niño(a) combinándola con (o además de) otros alimentos? | \|____\|<br>Si=1 ¿Cuánto tiempo en total? = \|____\|____\| meses<br>No=2<br>No sabe/ no contesta=9<br>[Utilice código 99 si no especifica tiempo en total] |

<table>
<tr><th colspan="2">8. Sección de atención al parto (APLIQUE A MUJERES QUE REFIEREN TENER UN HIJO MENOR DE 1 AÑO (SI ES UNA MUJER EMBARAZADA PRIMERIZA PASE A LA SECCIÓN 11)<br>ANOTE NOMBRE DEL <1 AÑO BENEFICIARIO DE OPORTUNIDADES=___________________________________<br>Ahora me gustaría hablar con Usted sobre la atención al parto que tuvo con [NOMBRE]</th></tr>
<tr><td>8.1 ¿En dónde se atendió el parto de (NOMBRE)?<br><br>[No lea, ni sugiera, registre la que indique la encuestada]</td><td>Centro de Salud de SSA……………………………..1<br>Clínica u Hospital del IMSS Oportunidades…2 |___|<br>Hospital de la SSA…………………………………..3<br>Partera……………………………………………..4<br>Consultorio o clínica privada…………………..5<br>Otro =8 (especificar)________________________________<br>No sabe…………………………………………….9</td></tr>
<tr><td>8.2 ¿Cómo fue el parto de (NOMBRE)?</td><td>Cesárea=1<br>Normal o natural=2 |___|<br>Otro=8 (especifique) _________________________________<br>No sabe=9</td></tr>
<tr><td>8.3 ¿A los cuántos meses nació [NOMBRE]?</td><td>A término=1<br>Prematuro=2 |___|<br>Meses=3 (especifique)………… |___|___|<br>Semanas=4(especifique)……… |___|___|<br>No sabe=9</td></tr>
<tr><td>8.4 Cuando nació (NOMBRE), ¿En qué momento se lo entregaron?</td><td>Al momento=1<br>Antes de media hora ó 30 min. =2 |___|<br>Horas después=3 (especifique)……|___|___|<br>El mismo día, no sabe horas=4<br>Al otro día=5<br>Al tercer día=6<br>Otro =8 (especificar)_______________________<br>No sabe/no recuerda=9</td></tr>
<tr><td>8.5 Cuando nació [NOMBRE], ¿En dónde se quedó o estaba su hijo, mientras estuvieron en el hospital o clínica para la atención del parto?</td><td>Se quedó en la misma habitación con ella=1<br>Se quedó en el cunero=2 |____|<br>Requirió incubadora=3<br>Otro=8 (especifique)____________________________________<br>No sabe=9</td></tr>
<tr><td>8.6 ¿Qué opina sobre el alojamiento conjunto (que el bebé y la madre estén la misma habitación)?</td><td>Que está bien=1<br>No está bien=2 |____|<br>No sabe, no responde=9</td></tr>
<tr><td>8.7 ¿Por qué razón opina eso sobre el alojamiento conjunto (que el niño(a) se quedé con la madre en la misma habitación)?</td><td>Porque lo puede cuidar mejor=1<br>Se puede alimentar con pecho al bebé=2 |____|<br>Para tener contacto con la mamá=3<br>Por seguridad=4<br>Prefiere que lo vigile la enfermera o médico=5<br>Lo hacen porque las enfermeras tienen mucho trabajo=6<br>Es más inseguro=7<br>Otro=8 (especifique)_______________________<br>No sabe=9</td></tr>
<tr><td>8.8 ¿Usted le dio pecho a (NOMBRE), mientras estuvo en el hospital o clínica para la atención al parto?</td><td>Si=1 |____|<br>No=2 →Pase a 8.15</td></tr>
<tr><td>8.9 ¿Al cuánto tiempo de nacido(a) [NOMBRE] le dio pecho por primera vez?</td><td>Al momento=1 |____|<br>Antes de la media hora ó 30 min.=2<br>Cuándo se lo entregaron=3 (especifique núm. horas) |___|___|<br>El mismo día, no sabe horas=4<br>Al otro día=5<br>Al tercer día=6<br>Otro =8 (especificar)__________________________________<br>No sabe/no recuerda=9</td></tr>
</table>

| | |
|---|---|
| 8.10 ¿Tuvo algún problema para dar pecho a (NOMBRE) mientras estuvo en el hospital o en los primeros días de nacido? | Si=1<br>No=2 } **Pase a 8.15**<br>No sabe=9 |____| |
| 8.11 ¿Qué problema tuvo para dar pecho a (NOMBRE) mientras estuvo en el hospital o en los primeros días de nacido?<br><br>*[REGISTRE LOS PRIMEROS TRES QUE MENCIONE]* | Dolor o pezones irritados o lastimados =01<br>Grietas en pezones=02 |___|___|<br>Leche no baja =03<br>No tuvo suficiente leche=04 |___|___|<br>Leche aguada o considerada de baja calidad=05<br>Pechos congestionados=06 |___|___|<br>Bebé no quería comer o agarrar el pecho=07<br>Bebé no sabía agarrar el pecho=08<br>La madre no quiso dar pecho=09<br>Otro=88 (especificar) ____________________________________<br>No sabe=99 |
| 8.12 De los problemas que me acaba de mencionar, ¿Cuál considera el más importante?<br><br>*[Si sólo menciona **una** repita el código]* | |____|____| |
| 8.13 ¿Le ayudó alguien a resolverlos? | Si=1 |____|<br>No=2 } **Pase a 8.15**<br>No sabe/no recuerda=9 |
| 8.14 ¿Quién le ayudó a resolver sus problemas cuando daba pecho a [NOMBRE]? | Médico=1<br>Enfermera o personal de salud=2 |____|<br>Madre =3<br>Suegra=4<br>Amiga, conocida=5<br>Vocal de Oportunidades=6<br>Otro=8 (especifique)_______________________________<br>No sabe=9 |
| 8.15 Durante su estancia en la clínica u hospital donde nació (NOMBRE) ¿Usted o alguien más **le dieron otros líquidos o leche** diferente a la leche materna a su bebé? | Si=1<br>No=2 } **Pase siguiente Sección** |____|<br>No sabe=9 |
| 8.16 ¿Qué le dio o le dieron (tés, líquidos u otra leche) a su bebé mientras estuvo en el hospital o clínica?<br><br>[No lea ni sugiera las respuestas. ANOTE LAS DOS MÁS IMPORTANTES] | Solución glucosada=01<br>Leche de fórmula=02 |____|____|<br>Leche Nido=03<br>Leche Liconsa=04 |____|____|<br>Leche Nutricrece=05<br>Otra leche=06 (especificar)____________________________<br>Agua natural=07<br>Té=08<br>Jugo de frutas o industrializado=09<br>Otro=88 (especificar)____________________________<br>No sabe=99 |
| 8.17 ¿En qué utensilio se lo dió o se lo dieron? | Mamila=1<br>Cuchara=2 |____|<br>Taza=3<br>Gotero/jeringa/sonda=4<br>Otro=8 (especificar)_________________________________<br>No sabe=9 |

| | |
|---|---|
| 8.18 ¿Cuál fue la principal razón por la que se lo dió o le dieron otros líquidos o leche diferente a la leche materna a NOMBRE? | Tenía sed=01<br>Tenía cólicos=02<br>Tenía estreñimiento=03 \|____\|____\|<br>Tenía hambre=04<br>Lloraba mucho=05<br>Diarrea=06<br>Bebé no agarró o no aceptó el pecho=07<br>Cesárea o parto complicado=08<br>Toma de medicamentos madre=09<br>Otro=88 (especificar) ___________________________________<br>No sabe=99 |
| 8.19 ¿Quién le recomendó o aconsejó que se lo diera? | Medico=1<br>Enfermera=2 \|____\|<br>Esposo=3<br>Suegra=4<br>Madre=5<br>Nadie, ella misma=6<br>Otra=8 (especificar) ___________________________________<br>No sabe=9 |
| | |

| | |
|---|---|
| **Sección 9. Prácticas de alimentación infantil para niños menores de 24 meses.**<br><br>**Leer: "Ahora me gustaría que platicaramos sobre la alimentación de (NOMBRE)".** | |
| 9.1 ¿Aún le da pecho a (NOMBRE)? | Si …….1 \|____\|<br>No ……2 → **Pase a 9.6** |
| 9.2 ¿Ayer le dio pecho a (NOMBRE) durante el día o la noche? | Si …….1<br>No ……2 → **Pase a 9.5** \|____\|<br>No sabe / No se acuerda……9 → **Pase a 9.5** |
| 9.3 ¿Cuántas veces le dio pecho ayer en el **DÍA** (desde que Usted se levantó hasta que Usted se fue a dormir)?<br><br>**[AYUDE A LA MADRE A RECORDAR]** | \|____\|____\| # Tetadas ayer de **DÍA**<br>No sabe / No se acuerda ……………………99 |
| 9.4 ¿Cuántas veces le dio pecho **ANOCHE** (desde que Usted se fue a dormir hasta que Usted se levantó en la mañana)?<br><br>**[AYUDE A LA MADRE A RECORDAR]** | \|____\|____\|# Tetadas de **ANOCHE**<br>No sabe / No se acuerda ……………………99 |
| 9.5 ¿Le da pecho a su bebé cada vez que lo pide? o sea, a **libre demanda**. | Si …….1<br>No ……2 \|____\|<br>No sabe / No se acuerda….. 9 |
| 9.6 ¿Le da la leche de su pecho en biberón (mamila)? | Si …….1<br>No ……2 \|____\|<br>No sabe / No se acuerda….. 9 |
| 9.7 ¿Cuántos tiempo en total dio o ha dado pecho a (NOMBRE)?<br><br>(Si menos de 1 mes, anote los días. Si más de 1 mes, anote los meses (si menos de 10, anote 0 y luego el #). Cuando la Mamá haya amamantado más de 1 año, convierta a meses: *(Ejemplos: 1año=12 m; 2 años=24 m; 3 años=36 m; 4 años=48 m)*<br>***NOTA al ENCUESTADOR: NO redondee los meses, sólo registre meses cumplidos*** | \|____\|____\| ó \|____\|____\|<br>DÍAS MESES<br><br>NO SABE / NO SE ACUERDA ……..99 |
| 9.8 ¿Usted cree que haya que limpiar el pecho de la madre antes de dar leche a su bebé? | Si=1<br>No=2 } **Pase a 9.10** \|____\|<br>No sabe=9 |

| | |
|---|---|
| 9.9 ¿Por qué cree que se tiene que limpiar el pecho de la madre antes de dar leche a su bebé? | Porque si está sucio se puede enfermar=1<br>Porque el sabor de la leche materna cambia=2 \|____\|<br>Porque el médico lo recomienda=3<br>Porque la enfermera lo recomienda=4<br>Porque la suegra se lo recomienda=5<br>Otro=8 (especificar)____________________________<br>No sabe=9 |
| 9.10 ¿Usted qué entiende por empacho del niño(a) o (NOMBRE)?<br><br>**[Utilice código 1=Si; 2=No; 9=No sabe]**<br><br>*[Todas las casillas deberán tener dato]* | 01. Dolor del estómago……………………………… \|___\|<br>02. Inflamación del estómago………………………… \|___\|<br>03. Cólicos……………………………………………… \|___\|<br>04. Diarrea……………………………………………… \|___\|<br>05. Estreñimiento…………………………………… \|___\|<br>06. Lengua blanca o amarilla………………………… \|___\|<br>07. Baba blanca ……………………………………… \|___\|<br>08. Llanto, tristeza e inquietud……………………… \|___\|<br>09. Gases, reflujo…………………………………… \|___\|<br>10. Mucha sed………………………………………… \|___\|<br>88. Otro (esp)_________________________.........\|___\| |
| 9.11 ¿Usted por qué cree que ocurre el empacho?<br><br>**[Utilice código 1=Si; 2=No; 9=No sabe]**<br><br>*[Todas las casillas deberán tener dato]* | 1. Por un susto………………………………………… \|___\|<br>2. Cuando se sobrecarga el estómago……………… \|___\|<br>3. Cuando se ha tomado demasiada leche o alimento en particular……………………………… \|___\|<br>4. Cuando se dan alimentos no bien cocidos (o indigeribles) ………………………………………. \|___\|<br>5. Por ofrecer alimentos fuera del horario (antes o después de dormir) ……………………………………………… \|___\|<br>6. Por dar alimentos secos………………………………. \|___\|<br>8. Otro (esp)__________________________ ....... \|___\| |
| 9.12 De sus actividades cotidianas. ¿Qué cree que puede afectar su leche y le pueda hacer daño a su bebé?<br><br>**[Utilice código 1=Si; 2=No; 9=No sabe]**<br><br>*[Todas las casillas deberán tener dato]* | 1. Tomar baño y dar pecho……………………………… \|___\|<br>2. Llegar de la calle y darle pecho……………………. \|___\|<br>3. Tener un susto y dar pecho………………………… \|___\|<br>4. Cuando el cuerpo está caliente y da pecho…… \|___\|<br>5. Si come alimentos fríos y da pecho………………. \|___\|<br>8. Otro (esp)____________________________..... \|___\| |
| **Leer: "Señora ahora vamos a hablar sobre lo que (NOMBRE) comió o se alimentó el día de ayer".** | |
| **A** | **B** |
| 9.13 **Alimentos** | ¿En cualquier momento de ayer ó de anoche le dio a (NOMBRE) algunos de los siguientes alimentos? |
| a. Agua simple | Si…..1 No….2 \|__\|<br>No sabe/no recuerda… 9 |
| b. Suero oral,<br>c. Gotas, jarabes medicinales (vitaminas, minerales, medicinas) | Si…..1 No….2 \|__\|<br>Si…..1 No….2 \|__\|<br><br>No sabe/no recuerda… 9 |
| d. Fórmula infantil<br>e. Leche en polvo<br>f. Otra leche | Si…..1 No….2 \|__\| ¿Cuál?_______________________\|___\|<br>Si…..1 No….2 \|__\| ¿Cuál?_______________________\|___\|<br>Si…..1 No….2 \|__\| ¿Cuál?_______________________\|___\|<br>No sabe/no recuerda… 9<br><br>[El código de la leche se agregará en captura] |
| g. Leche LICONSA en polvo<br>h. Leche LICONSA líquida | Si…..1 No….2 \|__\|<br>Si…..1 No….2 \|__\|<br><br>No sabe/no recuerda… 9 |

| | |
|---|---|
| 9.14 **Líquidos no nutritivos**<br>a. Agua endulzada<br>b. Té (simple ó endulzado<br>c. Café con agua (simple ó endulzado)<br>d. Refresco<br>e. Caldo de fríjol (sólo el caldo)<br>f. Caldo de pollo ó res (sólo el caldo)<br>g. Aguamiel (sin fermentar)<br>h. Jugos de frutas | <br>Si.....1 No....2 \|__\|<br>Si.....1 No....2 \|__\|<br>Si.....1 No....2 \|__\|<br>Si.....1 No....2 \|__\|<br>Si.....1 No....2 \|__\|<br>Si.....1 No....2 \|__\|<br>Si.....1 No....2 \|__\|<br>Si.....1 No....2 \|__\|<br>No sabe/no recuerda… 9 |
| 9.**15 Líquidos nutritivos**<br>a. Atole con agua<br>b. Atole con leche<br>c. Otro cereal (como avena, tapioca, etc.) (Con agua o con leche) | <br>Si.....1 No....2 \|__\|<br>Si.....1 No....2 \|__\|<br>Si.....1 No....2 \|__\|<br>No sabe/no recuerda… 9 |
| **A** | **B** |
| Alimentos | ¿En cualquier momento de ayer ó de anoche le dio a (NOMBRE) algunos de los siguientes alimentos? |
| 9.16 **Cereales y leguminosas**<br>a. Sopa de pasta<br>b. Arroz<br>c. Tortillas<br>d. Cereales industrializados para bebés en caja<br>e. Pan (cualquiera)<br>f. Avena<br>g. Lentejas<br>h. Habas | <br>Si.....1 No....2 \|__\|<br>Si.....1 No....2 \|__\|<br>Si.....1 No....2 \|__\|<br>Si.....1 No....2 \|__\|<br>Si.....1 No....2 \|__\|<br>Si.....1 No....2 \|__\|<br>Si.....1 No....2 \|__\|<br>Si.....1 No....2 \|__\|<br>No sabe/no recuerda… 9 |
| i. Frutas<br>j. Verduras<br>(Incluye envasados ó en frasco. NO DIGA MARCAS) | Si.....1 No....2 \|__\| ¿Cuáles?______________________\|_____\|<br>______________________\|_____\|<br>______________________\|_____\|<br>Si.....1 No....2 \|__\| ¿Cuáles? ______________________\|_____\|<br>______________________\|_____\|<br>______________________\|_____\|<br>No sabe/no recuerda… 9<br>[Cuáles frutas y verduras, registre las tres principales, el código se asignará en captura] |
| k. Carne de res, cerdo.<br>l. Corazón, hígado, menudencias (riñón, mollejas, etc.)<br>(Incluye envasados ó en frasco. NO DIGA MARCAS) | Si.....1 No....2 \|__\|<br>Si.....1 No....2 \|__\|<br>No sabe/no recuerda… 9 |
| m. Carne de pollo<br>n. Pescado (fresco ó en lata)<br>o. Pancita<br>(Incluye envasados ó en frasco. NO DIGA MARCAS) | Si.....1 No....2 \|__\|<br>Si.....1 No....2 \|__\|<br>Si.....1 No....2 \|__\|<br>No sabe/no recuerda… 9 |
| p. Jamón ó salchichas.<br>q. Otros embutidos<br>(Incluye envasados ó en frasco. NO DIGA MARCAS) | Si.....1 No....2 \|__\|<br>Si.....1 No....2 \|__\|<br>No sabe/no recuerda… 9 |
| r. Huevo | Si.....1 No....2 \|__\|<br>No sabe/no recuerda… 9 |

| | |
|---|---|
| s. Queso (cualquiera)<br>t. Yakult ó similares<br>u. Yogurt<br>v. Tipo danoninos | Si…..1 No….2 \|__\|<br>Si…..1 No….2 \|__\|<br>Si…..1 No….2 \|__\|<br>Si…..1 No….2 \|__\|<br>No sabe/no recuerda… 9 |
| w. Papilla del programa OPORTUNIDADES<br>x. Polvitos de OPORTUNIDADES (micronutrimentos en polvo) | Si…..1 No….2 \|__\|<br>Si…..1 No….2 \|__\|<br>No sabe/no recuerda… 9 |
| 9.17 **MISCELÁNEOS**<br>a. Frituras (totis, chicharrones, chetos, etc)<br>b. Galletas, dulces ó pastelitos (gansito, donas, etc.) | Si…..1 No….2 \|__\|<br>Si…..1 No….2 \|__\|<br>No sabe/no recuerda… 9 |
| 9.18 ¿En el día de ayer ó anoche (NOMBRE) tomó algún alimento en biberón como jugos, cereales, te ó infusiones u otros, sin contar leche materna? | Si…..1 No….2 \|__\|<br>No sabe/no recuerda… 9 |

| **10. Sección consejería sobre alimentación en consulta de revisión al niño(a)<br>[APLIQUE A MUJERES ENCUESTADAS CON UN HIJO MENOR DE 6 MESES ó 1 AÑO. NOMBRE=____________________________<br>Leer: "Ahora me gustaría hablar sobre la alimentación de su bebé o de [NOMBRE] en los primeros meses de vida".** | | |
|---|---|---|
| 10.1 ¿A los cuántos días después de nacido llevó a (NOMBRE) a consulta de revisión o de atención? | A los 3 días =1<br>A los 7 días =2<br>A los 14 días =3<br>Al mes =4<br>Otro(especificar)=8_______________________________<br>No sabe=9 | \|____\| |
| 10.2 ¿Le dijeron algo sobre alimentación del menor cuando llevó al niño(a) a consulta (cualquiera)? | Si=1<br>No=2 } **Pase a 10.4**<br>No sabe=9 | \|____\| |
| 10.3 ¿Qué le dijeron sobre alimentación del menor cuando llevó al niño(a) a consulta?<br>*[No lea, ni sugiera las respuestas]* | a. Darle té, agua<br>b. Dar leche materna exclusiva<br>c. Que le diera pecho (no exclusiva)<br>d. Que le diera fórmula<br>e. Probaditas de alimentos<br>f. Alimentarlo cada 3 horas<br>g. Limpiarse el pecho<br>h. Otro (especificar)______________________ | Si=1 No=2 \|____\|<br>Si=1 No=2 \|____\|<br>Si=1 No=2 \|____\|<br>Si=1 No=2 \|____\|<br>Si=1 No=2 \|____\|<br>Si=1 No=2 \|____\|<br>Si=1 No=2 \|____\|<br>Si=1 No=2 \|____\|<br>**9=No sabe/ No menciona** |
| 10.4 En el Centro de Salud donde lleva a su niño a control o revisión ¿Le recomendaron o (le recomiendan) dar a su hijo(a) además de la leche materna alguna otra? | Si=1<br>No=2 } **Pase a 10.11**<br>No sabe/no contesta=9 | \|____\| |
| 10.5 ¿Quién principalmente le recomienda o recomendó dar otra leche o alimento diferente a la leche materna? | Médico……………………………………01<br>Enfermera………………………………02<br>Promotor de salud……………………..03<br>Auxiliar en salud………………………04<br>Vocal de Oportunidades…………….05<br>Partera……………………………………06<br>Pediatra (especialista)……………….07<br>Otro=88 (especificar)___________________________________<br>No sabe……………………………………99 | \|___\|___\| |

| | |
|---|---|
| 10.6 ¿A partir de qué edad le aconsejaron dar a su hijo(a) otra leche en el centro de salud? | \|____\|____\| meses<br><br>99= No sabe, no recuerda |
| 10.7 ¿Cuál fue la principal razón por la que le recomendaron (o recomiendan) dar a su hijo(a) además de la leche materna alguna otra leche o alimento?<br><br>[REGISTRE LOS PRIMEROS TRES QUE MENCIONE] | Cesárea =01<br>Toma de medicamentos de la madre=02 \|___\|___\|<br>Enfermedad de la madre (ej. Varicela)=03<br>Reflujo del niño=04<br>Intolerancia a la lactosa=05 \|___\|___\|<br>Diarrea=06<br>No tenía suficiente leche o no nutritiva=07<br>El niño queda con hambre, mi leche no lo llenaba=08<br>Complicaciones post-operatorias=09 \|___\|___\|<br>Tabaquismo=10<br>Niño(a) prematuro=11<br>Estreñimiento=12<br>Otro (especificar)=88 ______________________________<br>No sabe=99 |
| 10.8 ¿Qué tipo de leche o fórmula le recomendaron en el Centro de Salud? | Liconsa=1<br>Nido=2 \|___\|<br>Nan, Enfamil, Enfagrow, Nursoy, etc. =3<br>Leche Lala, Alpura, etc.=4<br>Otro=8 (esp)___________________________<br>No sabe/ no recuerda=9 |
| 10.9 ¿Usted siguió o sigue la recomendación que le dieron en el Centro de Salud sobre darle otra leche diferente a la materna? | Si=1 → **Pase a 10.11**<br>No=2 \|___\|<br>No sabe/no contesta=9 |
| 10.10 ¿Por qué no sigue o no siguió la recomendación sobre darle otra leche? | Porque es mejor darle pecho…………………..1<br>Porque no tiene dinero…………………………..2 \|___\|<br>Porque quiere darle amor a su hijo(a)…….3<br>Porque su madre le dijó lo contrario……….4<br>Porque no cree en médico……………………..5<br>Otro=8 (especificar)____________________________________<br>No sabe…………………………………………………..9 |
| 10.11 En el Centro de Salud donde lleva a su niño a control o revisión ¿Le recomendaron o (le recomiendan) dar a su hijo(a) probaditas de otros alimentos además de la leche materna? | Si=1<br>No=2 } **Pase a 10.16** \|___\|<br>No sabe/no contesta=9 |
| 10.12 A partir de qué edad, en el Centro de Salud, le aconsejaron (o aconsejan) dar a su hijo(a) probaditas de otros alimentos además de la leche materna? | \|____\|____\| meses<br>99= No sabe, no recuerda |
| 10.13 ¿Qué tipo de alimentos (aunque sea probaditas) le recomendaron dar a su hijo(a) además de la leche materna en el Centro de Salud? | Tortilla, pan, papa, arroz, etc.=1<br>Frutas=2 \|___\|<br>Verduras=3<br>Huevo, carne, hígado, pollo=4<br>Queso, yogurt, etc.=5<br>Frijol, haba, alubia, etc.=6<br>Gerber o alimentos industrializados=7<br>Otro (esp)___________________________ =8<br>No sabe=9 |
| 10.14 ¿Ud. siguió la recomendación que le dieron en el Centro de Salud sobre darle probaditas de otros alimentos a su hijo(a)? | Si=1 → **Pase a 10.16**<br>No=2 \|___\|<br>No sabe/no recuerda=9 |

| | |
|---|---|
| 10.15 ¿Por qué no sigue o no siguió la recomendación de que le dieron en el Centro de Salud? | Porque es mejor darle pecho hasta los 6 meses…1<br>Porque no tiene dinero……………………………………2 \|____\|<br>Porque quiere darle amor a su hijo(a)………………3<br>Porque su madre le dijó lo contrario…………………4<br>Porque no cree en el médico……………………………5<br>Porque es muy pequeño para darle alimentos…..6<br>Otro=8 (especificar)___________________________________<br>No sabe……………………………………………………………9 |
| 10.16 Alguien de sus familiares, amigas o conocidas, ¿le aconsejó (o aconseja) dar a su hijo(a) además de la leche materna alguna otra leche? | Si=1<br>No=2 } **Pase a 10.23** \|____\|<br>No sabe/ no contesta=9 } |
| 10.17 ¿Quién principalmente le aconsejó (o aconseja) dar a su hijo(a) además de la leche materna alguna otra leche? | Madre=1<br>Suegra=2 \|___\|___\|<br>Hermana(o)=3<br>Esposo/pareja=4<br>Amiga(o)=5<br>Abuela(o)=6<br>Otro=8 (especificar) ________________________<br>No sabe=9 |
| 10.18 ¿A partir de qué edad sus familiares/amigos le aconsejaron dar a su hijo(a) dar otra leche a su hijo? | \|____\|____\| meses<br><br>99= No sabe, no recuerda |
| 10.19 ¿Qué tipo de leche le aconsejaron sus familiares/amigos?<br><br>[No lea, ni sugiera las respuestas. Anote la más importante] | Fórmula ¿Cuál marca?_______________________....1<br>Nido……………………………………………………………………….2 \|____\|<br>Leche Liconsa…………………………………………………………3<br>Nutri-crece……………………………………………………………4<br>Mi leche nutrisano…………………………………………………5<br>Otra(especifique)______________________...........8<br>No sabe/no recuerda……………………………………………9 |
| 10.20 ¿Cuál fue la principal razón o motivo por la que su familiar, amigos o conocidos le aconsejaron darle otra leche al bebé aparte del pecho? | No tenía suficiente leche=1<br>Bebé prematuro o bajo de peso=2<br>Bebé se quedaba con hambre=3 \|____\|<br>Madre delgada o con bajo peso=4<br>El bebe no sabía agarrar el pecho=5<br>Leche de formula es mejor que la materna=6<br>El bebé se enferma(ó) (ej. estreñimiento, cólicos, infección, etc.)=7<br>Otro=8 (especificar) __________________________<br>No sabe=9 |
| 10.21 ¿Ud. siguió la recomendación de familiares/amigos sobre darle otra leche a su hijo(a)? | Si=1 → **Pase a 10.23**<br>No=2 \|____\|<br>No sabe/no recuerda=9 |
| 10.22 ¿Por qué no sigue o no siguió la recomendación de sus familiares/amigos? | Porque es mejor darle pecho………………………………1<br>Porque no tiene dinero…………………………………………2 \|____\|<br>Porque quiere darle amor a su hijo(a)…………………3<br>Porque su madre le dijo lo contrario……………………4<br>Porque no cree en lo que dicen sus familiares/amigos…..5<br>Otro=8 (especificar)___________________________________<br>No sabe………………………………………………………………9 |
| 10.23 Sus familiares/amigos ¿Le recomendaron o (le recomiendan) dar a su hijo(a) además de la leche materna algún alimento o probaditas de alimentos? | Si=1<br>No=2 } **Pase siguiente Sección** \|____\|<br>No sabe/no contesta=9 } |
| 10.24 A partir de qué edad, sus familiares/amigos, le aconsejaron (o aconseja) dar a su hijo(a) darle otros alimentos además de la leche materna? | \|____\|____\| meses<br><br>99= No sabe, no recuerda |

| | |
|---|---|
| 10.25 ¿Qué tipo de alimentos (aunque sea probaditas) le recomendaron dar a su hijo(a) además de la leche materna? | Tortilla, pan, papa, arroz, etc.=1<br>Frutas=2 \|____\|<br>Verduras=3<br>Huevo, carne, hígado, pollo=4<br>Queso, yogurt, etc.=5<br>Frijol, haba, alubia, etc.=6<br>Gerber o alimentos industrializados=7<br>Otro (esp)__________________________ =8<br>No sabe=9 |
| 10.26 ¿Ud. siguió la recomendación de familiares/amigos sobre darle probaditas de otros alimentos a su hijo(a)? | Si=1 → **Pase siguiente Sección**<br>No=2 \|____\|<br>No sabe/no recuerda=9 |
| 10.27 ¿Por qué no sigue o no siguió la recomendación de sus familiares/amigos? | Porque es mejor darle pecho hasta los 6 meses……1<br>Porque no tiene dinero…………………………………………2 \|____\|<br>Porque quiere darle amor a su hijo(a)……………….…3<br>Porque su madre le dijo lo contrario…………………….…4<br>Porque no cree en sus familiares/amigos…………….5<br>Porque cree que es muy pequeño para darle alimentos…..6<br>Otro=8 (especificar)___________________________________<br>No sabe………………………………………………………….....9 |

| | |
|---|---|
| **11. Sección Alimentación Complementaria (AC) [APLIQUE A TODAS LAS MUJERES] NOMBRE=_____________________________**<br><br>**Leer: "Ahora me gustaría comentar con Usted un poco más sobre la alimentación de su hijo(a)".** | |
| 11.1 ¿A partir de qué edad se pueden dar **otros líquidos** (tés, leches, agua, etc.) en la alimentación del bebé? | \|___\|___\| meses<br>No sabe o no responde= 99 |
| 11.2 ¿A partir de qué edad se pueden dar otros **alimentos sólidos o suaves** (ej. frutas, verduras, etc.) en la alimentación del bebé, además de la leche materna? | \|___\|___\| meses<br><br>No sabe o no responde= 99 |
| 11.3 ¿Cuáles alimentos sabe o ha escuchado que son los primeros en dar al niño(a) cuando comienza su alimentación con sólidos (además de la leche materna)?<br><br>**[Utilice código 1=Si; 2=No; 9=No sabe]**<br><br>*[Todas las casillas deberán tener dato]* | 01. Pan…………………………………………( )<br>02. Arroz………………………………………( )<br>03. Vegetales verdes……………….……( )<br>04. Frutas……………………………….……( )<br>05. Carne………………………………………( )<br>06. Órganos (ej. hígado de pollo) …( )<br>07. Pescado……………………………………( )<br>08. Huevo………………………………………( )<br>09. Oleaginosas (nueces, almendras, etc.) ……( )<br>10. Otras leches (de bote) …………………………..( )<br>11. Quesos o yogurt……………………………………..( )<br>88. Otro (esp) _______________________…….( )<br>99. No sabe…………………………………………………..( ) |
| 11.4 ¿Cuál cree Usted que es la consistencia o preparación adecuada para ofrecer los alimentos o líquidos al bebé los primeros meses de vida? | Molidos (papilla, pure)=1<br>Crudos=2 \|____\|<br>Cocidos=3<br>Líquidos=4<br>Picados o en trocitos=5<br>Otro =8 (especificar)_________________________<br>No sabe=9 |
| **[APLIQUE SI LA ENCUESTADA ES MADRE DE MENOR DE 1 AÑO Y SI REFIERE TENER PAREJA O ESPOSO]**<br>11.5 ¿Su pareja o esposo participa o se involucra en la alimentación de su hijo(a)? | Si=1<br>No=2 } **Pase siguiente Sección** \|____\|<br>No sabe=9 |
| 11.6 ¿A qué edad su pareja/esposo participó o se involucró más en la alimentación de su hijo(a)? | \|___\|____\|meses<br><br>No sabe/ no responde/no se involucra= 99 |

| **12. Sección Lactancia Materna Trabajadora**<br>**[ENCUESTADOR: APLIQUE A TODAS LAS MUJERES]**<br>**Leer: "Ahora me gustaría platicar sobre la alimentación del bebé cuando las mujeres trabajan o no pueden estar cerca de su bebé".** | |
|---|---|
| 12.1 ¿Usted trabaja o realiza actividades fuera del hogar que la mantienen la mayor parte del día lejos de su bebé? | Si=1<br>No=2 } **Pase a 12.7** \|___\|<br>No sabe=9 |
| 12.2 ¿Usted lleva a su hijo(a) con Usted a sus actividades fuera del hogar? | Si=1 → **Pase a 12.7**<br>No=2 \|___\|<br>No sabe=9 |
| 12.3 ¿Cuántos días de la semana realiza esas actividades fuera del hogar? | \|___\|número de días<br>Es variable = 8<br>No sabe = 9 |
| 12.4 ¿Cuántas horas en promedio por día se encuentra fuera de su hogar cuando realiza esas actividades? | \|___\|___\| número de horas<br>Es variable = 88<br>No sabe = 99 |
| 12.5 Cuando regrese al trabajo o a sus actividades fuera del hogar ¿Usted piensa seguir dando pecho a su bebé? | Si=1<br>No=2 \|___\|<br>No sabe=9 } **Pase a 12.7** |
| 12.6 Cuando regrese al trabajo o a sus actividades fuera del hogar ¿Por cuánto tiempo en total (en meses) planea (o planeó) darle pecho a su bebé?<br><br>[INDAGAR SOBRE CUALQUIER TIPO DE LACTANCIA MATERNA] | Total = \|___\|___\| meses<br><br>No sabe o no menciona = 99 |
| 12.7 ¿Usted cree que se pueda hacer algo para seguir dando pecho al bebé cuando una madre regresa a trabajar o está lejos de su hijo(a)? | Si=1<br>No=2 } **Pase a 12.9** \|___\|<br>No sabe=9 |
| 12.8 ¿Qué cree que se pueda hacer para seguir dando pecho al hijo(a) cuando la madre trabaja o está lejos de su hijo(a)? | Darle antes de irse o separarse=1 \|___\|<br>Darle cuando regresa a casa o vuelven a estar juntos=2<br>Extraerse la leche y guardarla=3<br>Todas las anteriores=4<br>Otro =8 (esp) ____________________________<br>No sabe=9 |
| 12.9 ¿Ud. Planea (o planeó) extraerse la leche para dejarla a su hijo(a) para cuando no esté cerca de él/ella?<br><br>[MUESTRE TARJETA CON OPCIONES DE RESPUESTA] | 1= No planea(ó)<br>2= Estoy pensando (o pensó) \|___\|<br>3= Está haciéndolo (o lo hizo)<br>4= Va a seguir haciéndolo (o lo hizo)<br>5= Otra respuesta diferente |
| **[APLIQUE SÓLO SI ES MADRE DE MENOR DE 1 AÑO (2 AÑOS) Y SI ESTÁ DANDO PECHO]**<br>12.10 ¿Usted se extrae la leche materna para dejarla a su hijo(a)? | Si=1<br>No=2 \|___\|<br>No sabe=9 |
| 12.11 ¿Alguien le ha aconsejado o enseñado sobre técnicas de extracción de leche materna para cuando no se está cerca del bebé? | Si=1<br>No=2 } \|___\|<br>No sabe=9 **Pase a 12.14** |
| 12.12 ¿Quién le ha comentado sobre técnicas de extracción de leche materna? | Médico=1<br>Enfermera o personal de salud=2 \|___\|<br>Madre=3<br>Suegra=4<br>Amiga, vecina, conocida=5<br>Vocal de Oportunidades=6<br>Abuela=7<br>Otro=8 (especificar)________________________<br>No sabe=9 |

| | |
|---|---|
| 12.13 ¿Cómo le recomendaron extraerse la leche materna?<br><br>[REGISTRE LAS PRIMERAS DOS OPCIONES DE RESPUESTA] | Manual o con bomba de extracción= 01 \|____\|____\|<br>Maquina de extracción=02<br>Otro=88 (especificar)______________________ \|____\|____\|<br>No sabe, no responde=99 |
| 12.14 Del 0 al 10, ¿qué tan confiada o segura está de que se pueda extraer la leche materna Usted misma?<br><br>[MUESTRE TARJETA CON ESCALA DE RESPUESTA] | \|____\|____\|<br><br>No sabe=99 |
| 12.15 Del 0 al 10, ¿qué tan segura o confiada está Usted de que sea útil sacarse la leche materna y ofrecerla al bebé?<br><br>[MUESTRE TARJETA CON ESCALA DE RESPUESTA] | \|____\|____\|<br><br>No sabe=99 |
| **[APLIQUE SI EN LA PREGUNTA ANTERIOR SU RESPUESTA FUE MENOR A 5, DE LO CONTRARIO PASE A 12.17]**<br><br>12.16 ¿Por qué cree que sea inútil (o no valga la pena) sacarse la leche materna para dejarla al niño(a)? | Con la de fórmula es suficiente=1<br>Una vez afuera ya no es nutritiva=2 \|____\|<br>Es muy poca=3<br>Se echa a perder (se contamina)=4<br>La de fórmula es el mismo alimento=5<br>El bebé no nota el cambio (sabor)=6<br>Otro(especificar)=8______________________<br>No sabe=9 |
| 12.17 ¿Usted cree que sea complicado extraerse la leche materna? | Si =1<br>No=2 } **Pase a 12.19** \|____\|<br>No sabe=9 } |
| 12.18 Del 0 al 10, ¿qué tan fácil cree que sea extraerse la leche materna?<br><br>[MUESTRE TARJETA CON ESCALA DE RESPUESTA] | \|____\|____\|<br><br>No sabe=99 |
| 12.19 ¿Ud. sabe por cuánto tiempo se puede **almacenar** la leche materna **fuera del pecho**? | Si =1<br>No=2 } **Pase a 12.21** \|____\|<br>No sabe=9 } |
| 12.20 ¿Cómo almacena y por cuánto tiempo sabe Usted o ha escuchado que se puede almacenar la leche materna **fuera del pecho**?<br><br>[REGISTRE HASTA TRES RESPUESTAS QUE MENCIONE] | No se puede conservar=1<br>\|____\|<br>A temperatura ambiente=2<br>¿Cuántas? \|___\|___\|días\| ___\|___\|horas \|____\|<br>En refrigeración=3<br>¿Cuántas? \|___\|___\|días \|___\|___\|horas \|____\|<br>Congelada=4<br>¿Cuántas? \|___\|___\|días \|___\|___\|horas<br><br>Otro (especificar)=8____________________________<br>No sabe=9 |
| **[APLIQUE SI LA ENCUESTADA REFIERE TENER PAREJA O ESPOSO, DE LO CONTRARIO PASE A SIGUIENTE SECCIÓN]**<br><br>12.21 ¿Usted cree que su **esposo/pareja** podría apoyarla para que Usted siga (o siguiera) dando leche materna al bebé y continúe con sus actividades fuera de casa? | Si =1 \|____\|<br>No=2 } **Pase siguiente Sección**<br>No sabe=9 } |
| 12.22 ¿Cómo cree que su **esposo/pareja** podría apoyarla para que siga dando leche materna al bebé aunque esté fuera de casa? | Con el cuidado de los hijos=1<br>Guía y recomendaciones sobre LM=2 \|____\|<br>Masajes, tés y alimentos que le ayuden=3<br>Darle espacio y tiempo para hacerlo=4<br>Llevándole al bebé a ratos para darle pecho=5<br>Darle la leche materna que deje la madre=6<br>Dejar de trabajar=7<br>Otro=8 (especificar) _________________________<br>No sabe=9 |

| 13. Sección Diarrea, Sueros de Rehidratación Oral (SRO) y lavado de manos.<br>[ESTA SECCIÓN APLICA A MADRES]<br>Leer: "Ahora me gustaría platicar con Usted sobre prácticas de higiene y sobre cambios en la alimentación cuando su hijo(a) se enferma". | |
|---|---|
| 13.1 ¿Usted cuenta con drenaje (descarga de aguas negras) en su hogar? | Si=1<br>No=2 \|____\|<br>No sabe=9 |
| 13.2 En su hogar, ¿cuál es la principal fuente o abastecimiento de agua? | Llave de la red pública de agua potable (dentro de casa)=1<br>Pozo=2 \|____\|<br>Cisterna que se llena con pipa=3<br>Depósito comunitario=4<br>Río o laguna=5<br>Otro=8 (especificar)________________________________<br>No responde=9 |
| 13.3 ¿En qué momento se lava Usted las manos cuando está en su casa?<br><br>**[Utilice código 1=Si; 2=No; 9=No sabe]**<br><br>*[Todas las casillas deberán tener dato]* | 01. Al momento de preparar los alimentos…………….( )<br>02. Antes de ir al baño…………………………………………( )<br>03. Después de ir al baño…………………………………….( )<br>04. Al momento de alimentar al niño <2años…………( )<br>05. Cuando regresa de la calle……………………………( )<br>06. Después de cambiar el pañal al niño(a)…………...( )<br>07. Cuando está en la cocina………………………………( )<br>08. Antes de consumir alimentos…………………………( )<br>88. Otro (especificar)________________________..( ) |
| 13.4 ¿Usted con qué se lava las manos?<br><br>[pídale que le muestre con que se lava las manos] | Con agua sola=1<br>Con agua y Jabón (cualquiera)=2 \|____\|<br>Desinfectante (yodo)=3<br>Vinagre=4<br>Cloro=5<br>Jabón y agua (combinado)=6<br>Alcohol o gel=7<br>Otro=8 (especificar) ____________________________<br>No responde=9 |
| 13.5 En los últimos tres meses, ¿Tuvo (NOMBRE) diarrea? | Si=1<br>No=2 } **Pase a 13.17** \|____\|<br>No sabe=9 |
| 13.6 Para Usted ¿cuándo una diarrea de (NOMBRE) es preocupante?<br><br>[REGISTRE HASTA TRES OPCIONES DE RESPUESTA] | Cuando hace muchas veces=01<br>Cuando hay sangrado=02 \|____\|____\|<br>Cuando no toma, ni come nada=03<br>Cuando el niño no deja de llorar=04 \|____\|____\|<br>Cuando el niño pierde peso=05<br>Cuando el niño tiene los labios secos=06 \|____\|____\|<br>Cuando el niño tiene los ojos tristes (hundidos)=07<br>Desde el principio (cuando tiene evacuaciones, se queja de dolor, etc.)=08<br>Otro (especificar)=88____________________<br>No sabe=99 |
| 13.7 De las opciones que me acaba de mencionar, para Usted ¿cuál es la más importante?<br>*[SI SÓLO MENCIONA **UNA** REPITA EL CÓDIGO]* | \|____\|____\| |
| 13.8 Ud. sabe o le han explicado, ¿Cuál fue la causa del último episodio de diarrea de [nombre]? | Si=1<br>No=2 } **Pase a 13.13** \|____\|<br>No sabe=9 |

| | |
|---|---|
| 13.9 ¿Cuál fue la causa del último episodio de diarrea de (NOMBRE)? | Parásitos intestinales (parasitosis)=1<br>Alimentos sucios e infectados=2 \|____\|<br>Falta de agua limpia=3<br>Contacto con piso y objetos sucios=4<br>Leche de fórmula=5<br>Contacto con animales o mascotas=6<br>Otro(especificar)=8____________________<br>No sabe=9 |
| 13.10 ¿Cuál fue el tratamiento para la diarrea de (NOMBRE) que le indicaron o recomendaron?<br><br>[REGISTRE HASTA TRES OPCIONES DE RESPUESTA] | Suero intravenoso=1<br>Desparasitación=2 \|____\|<br>Suero de rehidratación oral=3<br>Hidratación en el hogar (aguas, tés, caldos)=4 \|____\|<br>Continuar con lactancia materna=5<br>Medicamento (antibióticos, etc.)=6 \|____\|<br>Otro (especificar)=8____________________<br>No sabe=9 |
| 13.11 ¿Quién le indico el tratamiento en el último episodio de diarrea de (NOMBRE)? | Médico o enfermera=1<br>Madre o suegra=2 \|____\|<br>Conocido o vecino=3<br>Nadie, ella sola=4<br>Lo escuchó o vió en algún medio de comunicación=5<br>Otro=8 (esp) ________________________________<br>No sabe=9 |
| 13.12 ¿Por cuánto tiempo se lo indicaron o recomendaron? | \|___\|____\| días<br><br>No sabe o no recuerda = 99 |
| **Leer: "Ahora me gustaría que platicaramos como fue la alimentación de (NOMBRE) durante el episodio de diarrea".** | |
| 13.13 Durante el último episodio de diarrea (en los últimos tres meses), ¿le dió a (NOMBRE) lactancia materna? | Si=1 → **Pase a 13.15**<br>No=2 \|____\|<br>No sabe=9 |
| 13.14 ¿Por qué no le dió pecho a (NOMBRE) durante el último episodio de diarrea?<br><br>(REGISTRE HASTA TRES OPCIONES DE RESPUESTA)<br>**(AL TERMINAR PASE A 13.17)** | Porque así se lo indicaron en el Centro de Salud=1<br>Porque no aceptaba el pecho=2 \|____\|<br>Porque tenía sed=3<br>Porque tenía que tomar el tratamiento=4 \|____\|<br>Porque se quedó hospitalizado=5<br>Otro (especificar)=8____________________ \|____\|<br>No sabe=9 |
| 13.15 ¿Con qué frecuencia le dió lactancia materna a (NOMBRE) durante el último episodio de diarrea? | Igual=1<br>Menos=2 \|____\|<br>Más que antes de enfermarse=3<br>Otro (especificar)=8________________________<br>No sabe=9 |
| 13.16 ¿Por qué le dió pecho a (NOMBRE) con esa frecuencia durante el último episodio de diarrea? | Porque así lo pedía=1<br>Porque así se lo indicaron en el Centro Salud=2 \|____\|<br>Porque es bueno para el bebé=3<br>Porque no sabía qué hacer=4<br>Otro (especificar)=8________________________<br>No sabe, no responde=9 |
| 13.17 En el centro de salud ¿han desparasitado a (NOMBRE) en los últimos tres meses? | Si=1<br>No=2 } **Pase a 13.19** \|____\|<br>No sabe=9 |

| | |
|---|---|
| 13.18 ¿Cada cuando (nombre) ha recibido tratamiento de desparasitación? | Cada 3 meses=1<br>Cada 6 meses=2 \|____\|<br>Cada año=3<br>En la semana nacional de salud=4<br>Otro =8 (especificar) ___________________________<br>No sabe=9 |
| 13.19 ¿Usted pide o solicita que le den desparasitación a (NOMBRE)? | Si=1 → **Pase a 13.21**<br>No=2 \|____\|<br>No sabe=9 |
| 13.20 ¿Por qué no pide o solicita que desparasiten a (NOMBRE)? | Porque no lo necesita=1<br>Porque no hay en el centro de salud=2 \|____\|<br>Porque no le gusta al niño(a)=3<br>Porque ha tenido o tiene diarrea=4<br>Otro=8 (especificar) ____________________________<br>No sabe=9 |
| 13.21 ¿Usted usó o ha usado el suero de rehidratación Oral en el último episodio de diarrea?<br><br>**[Encuestador: puede ser mayor a 3 meses el tiempo del último episodio de diarrea]** | Si=1<br>No=2 }<br>No sabe=9 } **Pase a 13.23** \|____\| |
| 13.22 ¿Qué tipo de suero de rehidratación oral ha utilizado con (NOMBRE)?<br><br>**[AL TERMINAR PASE A 13.24]** | Casero=1<br>VIda suero oral (VSO)=2 \|____\|<br>Mezcla de bebidas carbonatadas (sidral+agua mineral)=3<br>Comercial (ej. Suerox, Pedialite)=4<br>Otro=8 (especifique) _______________________________<br>No sabe=9 |
| 13.23 ¿Por qué no ha usado suero de rehidratación oral? | Porque no lo necesita=1<br>Porque no hay en el centro de salud=2 \|____\|<br>Porque no le gusta al niño(a)=3<br>Porque fue una diarrea leve=4<br>Otro=8 (especifique) _______________________________<br>No sabe=9 |
| 13.24 ¿Usted sabe preparar **Vida Suero Oral (sobre de polvo)**? | Si=1<br>No=2 } \|____\|<br>No sabe=9 } **Pase a 13.26** |
| 13.25 ¿Cómo prepara Vida Suero Oral? | Disuelves el sobre en 1 lt de agua hervida =1<br>Otra=8 (especifique) ________________________ \|____\|<br>No sabe=9 |
| 13.26 ¿Usted sabe usar Vida Suero Oral? | Si=1<br>No=2 } \|____\|<br>No sabe=9 } **Pase a 13.28** |
| 13.27 ¿Cómo se usa **Vida Suero Oral**?<br><br>**[Utilice código 1=Si; 2=No; 9=No sabe]**<br><br>*[Todas las casillas deberán tener dato]* | 01. Se checa caducidad………………………………………………. \|___\|<br>02. Lo que no se consuma en 24 hrs se desecha……………. \|___\|<br>03. Se ofrece de forma continua hasta acabar la dosis (1/2 tacita para <6m y 1 tacita para <1)…………………………………………. \|___\|<br>04. Se da por cada evacuación ……………………………………. \|___\|<br>05. Se da por cada episodio de vómito………………………….\|___\|<br>06. Se alterna con lactancia materna ………………………….. \|___\|<br>07. Se introduce fórmula y otros líquidos……………………….\|___\|<br>08. Se ofrece a cucharadas o en tacita ………………………….. \|___\|<br>09. Se da cuando hace mucho calor ……………………………… \|___\|<br>88. Otro (especificar)_______________________ ………… \|___\| |
| 13.28 ¿Usted usa (o ha usado **biberones o mamilas** con sus hijo(a)(s) para dar cualquier tipo de líquidos o alimentos? | Si =1<br>No=2 } ***Pase a 13.32*** \|____\|<br>No sabe=9 } |

| | |
|---|---|
| 13.29 ¿Alguien le ha hablado sobre los riesgos de usar el biberón/mamila para alimentar a su hijo(a)? | Si=1<br>No=2<br>No sabe=9 } ***pase a 13.32*** \|____\| |
| 13.30 ¿Cuáles son los riesgos que conoce sobre el **uso de biberones/mamilas** para el niño(a)?<br><br>[REGISTRE HASTA TRES OPCIONES DE RESPUESTA] | Se acostumbra el bebé=01<br>Deforma o pica los dientes=02 \|___\|___\|<br>Ninguno, está bien usarlo=03<br>Reflujo y riesgo de ahogamiento=04 \|___\|___\|<br>Difícil lavado y guarda microorganismos =05<br>Mayor riesgo de diarreas=06 \|___\|___\|<br>Mayor riesgo de parasitosis=07<br>Se sobrealimenta (lo hace más grande o gordito)=08<br>Se puede ahogar porque sale mucho líquido=09<br>Les entra aire al estómago (cólicos)=10<br>Otro=88 (especificar)__________________________<br>No sabe=99 |
| 13.31 De lo que me acaba de mencionar, ¿Cuál considera lo más importante?<br>*[Si sólo menciona **una** repita el código]* | \|___\|___\| |
| 13.32 ¿Usted usa (o ha usado) **chupones** con sus hijo(a)(s) para dar cualquier tipo de líquidos o alimentos? | Si =1<br>No=2 } ***pase a 13.36***<br>No sabe=9 \|____\| |
| 13.33 ¿Usted sabe si el uso de **chupone**s puede tener riesgos para el bebé? | Si =1<br>No=2 } ***pase a 13.36***<br>No sabe=9 \|____\| |
| 13.34 ¿Cuáles riesgos conoce sobre el **uso de chupones** durante los primeros meses de vida?<br><br>[REGISTRE LAS PRIMERAS TRES OPCIONES QUE MENCIONÉ EL ENCUESTADO] | Se acostumbra al bebé=01<br>Deforma o pican los dientes=02 \|___\|___\|<br>Ninguno, está bien usarlo=03<br>Reflujo y riesgo de ahogamiento=04 \|___\|___\|<br>Difícil lavado y guarda microorganismos =05<br>Mayor riesgo de diarreas=06 \|___\|___\|<br>Mayor riesgo de parasitosis=07<br>Les entra aire al estómago (cólicos)=08<br>Otro=88 (especificar)____________________<br>No sabe=99 |
| 13.35 De lo que me acaba de mencionar, ¿Cuál considera lo más importante?<br>*[Si sólo menciona **una** repita el código]* | \|___\|___\| |
| **Leer: "Ahora quiero que me platique lo que hace en su hogar pare prevenir enfermedades del estómago".** | |
| 13.36 ¿Qué hace Usted para cuidar a sus hijo(a)(s) para que no se enfermen de diarrea?<br><br>[REGISTRE HASTA TRES OPCIONES DE RESPUESTA] | Lavado de manos=1<br>Lavado de frutas y verduras (agua y jabón)=2 \|____\|<br>Desinfección de frutas y verduras (ej. con yodo)=3<br>Hervor de agua y/o leche=4 \|____\|<br>Cocimiento de carnes y pescados=5<br>Alimentos refrigerados=6 \|____\|<br>Cubrir alimentos y almacenar=7<br>Otro=8 (especificar) __________________________<br>No sabe=9 |
| 13.37 De lo que me acaba de mencionar, para Usted ¿qué es lo más importante?<br>*[Si sólo menciona **una** repita el código]* | \|____\| |

| **14. Sección salud física y mental y valores de la mujer embarazada o madre del menor de 2 años**<br>**[APLICAR A TODAS LAS MUJERES]**<br>**"Ahora me gustaría comentar un poco con Usted sobre cómo se encuentra en la actualidad, en relación a su salud física".** | |
|---|---|
| 14.1 ¿Cómo considera su peso actual? | Bajo peso=1<br>Normal=2 \|____\|<br>Sobrepeso=3<br>Con obesidad=4<br>Otro=8 (especificar) ________________________<br>No sabe=9 |
| **[APLIQUE SI LA ENCUESTADA REFIERE TENER PAREJA O ESPOSO, DE LO CONTRARIO PASE A PREG. 14.4]**<br><br>14.2 ¿Le comenta algo su pareja o esposo sobre su cuerpo o como se ve? | Si=1<br>No=2 } \|____\|<br>No sabe=9 } **Pase a 14.4** |
| 14.3 ¿Qué le comenta su **pareja /esposo**?<br><br>**[Utilice código 1=Si; 2=No; 9=No sabe]**<br><br>*[Todas las casillas deberán tener dato]* | 1. Que estoy con bajo peso……………………\|___\|<br>2. Que tengo que ganar más peso………….\|___\|<br>3. Que estoy bien……………………………………\|___\|<br>4. Que tengo que hacer ejercicio…………….\|___\|<br>5. Que me debo de cuidar……………………..\|___\|<br>6. Que estoy ganando peso…………………….\|___\|<br>8. Otro (especificar)_________________ \|___\| |
| 14.4 ¿Cuál es su peso actual (auto-reporte)? | Peso \|___\|___\|___\|.\|___\|kgs<br><br>No sabe/no recuerda=999.9 |
| **Encuestador: Este renglón se contesta por observación de Usted".** | |
| 14.4.1- Sobrepeso aparente<br>14.4.2- Obesidad aparente<br>**[Utilice código 1=Si; 2=No; 9=No sabe]** | \|___\|<br>\|___\| |
| 14.5 ¿Algún médico le ha diagnosticado alguna enfermedad crónica? | Si=1<br>No=2→ **Pase a 14.9** \|____\| |
| 14.6 ¿Cuál enfermedad le han diagnosticado?<br><br>**[Utilice código 1=Si; 2=No; 9=No sabe]**<br><br>*[Todas las casillas deberán tener dato]* | Hipertensión o presión alta=1 ……………………………….( )<br>Diabetes o azúcar alta en sangre=2 ………………………..( )<br>Colesterol alto=3……………………………………………………( )<br>Triglicéridos altos=4 ……………………………………………..( )<br>Otro =8 (especificar) _________________________ ( )<br>No sabe=9 ……………………………………………………………….( ) |
| 14.7 ¿Actualmente está tomando algún medicamento para bajar o controlar su enfermedad? | Si=1<br>No=2→ **Pase a 14.9** \|____\| |
| 14.8 ¿Cómo se llama el medicamento?<br><br>[Solicite que se lo muestren, si es posible]<br><br>**(NO SABE /NO RECUERDA= escribir en la línea NINGUNO)** | 1 = (esp) ___________________________<br><br>2= (esp) ___________________________<br><br>3 = (esp) ___________________________<br><br>4 = (esp) ___________________________<br><br>5 = (esp) ___________________________ |

<table>
<tr><th colspan="2">Leer: “Por último, me gustaría que platicáramos sobre lo que más valora en la vida o lo que le angustia”.<br><br>[NO LEER, NI SUGERIR RESPUESTAS, REGISTRE LA(S) QUE MENCIONE EL ENCUESTADO]</th></tr>
<tr><td>14.9 ¿Qué es lo más importante para Usted en general (familia, la vida)?<br><br>[Utilice código 1=Si; 2=No; 9=No sabe]<br><br>[Todas las casillas deberán tener dato]</td><td>1. La salud…………………..…………….…......|___|<br>2. La seguridad……………..………………........|___|<br>3. Mejoría en cuestiones económicas..........|___|<br>4. La familia……………………………………….|___|<br>5. Lo(s)(as) hijos(as).....................................|___|<br>6. Trabajo .....................................................|___|<br>7. Educación de los hijos(a)(s)…………………..|___|<br>8. Otro(especificar)________________......|___|</td></tr>
<tr><td>14.10 ¿Qué es lo más importante para Usted con respecto a su bebé (hijo(a) <2 años)?<br><br>[Utilice código 1=Si; 2=No; 9=No sabe]<br><br>[Todas las casillas deberán tener dato]</td><td>01. Seguridad…………………………………......|___|<br>02. Que crezca sano y fuerte……………………|___|<br>03. Que no se enferme……………………..……|___|<br>04. Que sea inteligente………………….….......|___|<br>05. Que vaya a la escuela………………….……|___|<br>06. Que no los maltraten (violencia domestica)…….|___|<br>07. Su salud……………………………….............|___|<br>08. Su alimentación……………..……………….|___|<br>09. Su desarrollo (ej. habla)……………………..|___|<br>88. Otro(especificar)________________.....|___|</td></tr>
<tr><td>14.11 ¿Qué es lo más importante para Usted como persona, como mujer?<br><br>[Utilice código 1=Si; 2=No; 9=No sabe]<br><br>[Todas las casillas deberán tener dato]</td><td>1. Estar sana……………………………….……….|___|<br>2. No ser una carga para mi familia………....|___|<br>3. No enfermarme para no gastar…………….|___|<br>4. Sentirse bien (auto-estima)……………………|___|<br>5. Que no me maltraten (violencia doméstica)….….|___|<br>6. Poder cuidar a mi familia………………………|___|<br>8. Otro (especificar)________________......|___|</td></tr>
<tr><td>14.12 ¿Qué hace Usted para mantener su salud?<br><br>[Utilice código 1=Si; 2=No; 9=No sabe]<br><br>[Todas las casillas deberán tener dato]</td><td>01. Hacer ejercicio……………………………………|___|<br>02. Comer saludable (ej. comer frutas y verduras)...|___|<br>03. No comer comida chatarra (ej. frituras, etc.)……|___|<br>04. No tomar refresco…………………………….|___|<br>05. Estar sana…………………………………………|___|<br>06. No ser una carga para mi familia………..|___|<br>07.No enfermarme para no gastar..………….|___|<br>08. Checarse frecuentemente con el médico….…...|___|<br>88. Otro (especificar)________________...|___|</td></tr>
<tr><td>14.13 ¿Qué le pone triste o le angustia con respecto a su familia o hijos?<br><br>[Utilice código 1=Si; 2=No; 9=No sabe]<br><br>[Todas las casillas deberán tener dato]</td><td>01. Seguridad……………………………….........|___|<br>02. Preocupaciones económicas……………..|___|<br>03. Dejar a sus hijos solos………………..……..|___|<br>04. No ser buena madre/esposa……………..|___|<br>05. Que no se enferme(n)……………………....|___|<br>06. No tener para darles de comer…………..|___|<br>07. Educación de su(s) hijo(a)(s)……………..|___|<br>08. Dar (les) estabilidad familiar……………..|___|<br>09. Que no los maltraten (violencia domestica)……|___|<br>88. Otro (especificar)________________..|___|</td></tr>
</table>

| | |
|---|---|
| 14.14 ¿Qué le puede motivar a Usted si se le pide que haga algún **cambio** para mejorar su alimentación?<br><br>**[Utilice código 1=Si; 2=No; 9=No sabe]**<br><br>*[Todas las casillas deberán tener dato]* | 1. Mejorar salud……………………………….….\|___\|<br>2. Mi peso corporal……………………………….\|___\|<br>3. Apariencia física……………………………….\|___\|<br>4. Por enfermedad………………………………...\|___\|<br>5. Mejor economía………………………..………\|___\|<br>6. Salud de su familia…………………….……….\|___\|<br>7. Sentirse bien (auto-estima)…………………\|___\|<br>8. Otro (especificar)_________________...\|___\| |
| 14.15 ¿Qué **podría impedir** que Usted y/o su familia lleven una alimentación saludable?<br><br>**[Utilice código 1=Si; 2=No; 9=No sabe]**<br><br>*[Todas las casillas deberán tener dato]* | 01. Nada……………………………………….……\|___\|<br>02. Costos……………………………………….....\|___\|<br>03. Preparación complicada…………………..\|___\|<br>04. Recomendaciones difíciles de cumplir….……...\|___\|<br>05. No le(s) gustan las verduras…………….\|___\|<br>06. No tiene disponibles alimentos sanos……………\|___\|<br>07. No sabe qué comer…………………………\|___\|<br>08. El gusto por ciertos alimentos…….…..\|___\|<br>09. No tener tiempo para preparar los alimentos ella misma…………………………………………….\|___\|<br>88. Otro (especificar)____________........\|___\| |
| **[APLIQUE A MUJERES EMBARAZADAS DE CONSULTA PRENATAL Y MADRES DE NIÑOS MENORES DE 6 MESES]** | |
| 14.16 Durante el último mes, ¿ha estado molesta a menudo o frecuentemente por sentirse triste, deprimido o sin esperanza? | Si=1<br>No=2 \|___\|<br>No sabe=9 |
| 14.17 Durante el último mes, ¿a menudo se ha sentido molesta por tener poco interés o placer en hacer las cosas? | Si=1<br>No=2 \|___\|<br>No sabe=9 |
| **[Aplique si respondió "Si" a cualquiera de las dos preguntas anteriores]**<br>14.18 Con respecto a lo que Usted siente, ¿necesita o quiere ayuda? | Si=1 \|___\|<br>No=2<br>No sabe=9 |

| **15.1 IDENTIFICADOR DE LA PAREJA/ESPOSO DE LA MUJER ENCUESTADA**<br>**15.2 IDENTIFICADOR DEL CUESTIONARIO DE LA PAREJA/ESPOSO DE LA MUJER ENCUESTADA** |
|---|
| Folio de la persona: ……………………………………\|___\|___\|___\|___\|___\|<br><br>Número progresivo del cuestionario:…………………………….\|___\|___\|___\|___\|<br><br>**ANOTE 99999 EN CASO QUE NO CORRESPONDA CON NINGÚN HOMBRE ENCUESTADO.** |

| OBSERVACIONES |
|---|
| |
| |
| |
| |
| |
| |

## EVALUACIÓN DE LA INFORMACIÓN

| PREGUNTA | RESPUESTA | |
|---|---|---|
| DURANTE LA ENTREVISTA, ¿LA INFORMANTE ENTENDIÓ A LAS PREGUNTAS QUE USTED LE HIZO? | SI.........................1<br>NO.......................2 | \|___\| |
| ¿QUÉ TAN CONFIABLES LE PARECIERON LAS RESPUESTAS DE LA ENTREVISTADA? | MUY CONFIABLES..........1<br>REGULAR.........................2<br>POCO CONFIABLES........3 | \|___\| |
| DURANTE EL TRANSCURSO DE LA ENTREVISTA, LA ENTREVISTADA SE MOSTRÓ: | INTERESADO.......................1<br>POCO INTERESADO............2<br>NO INTERESADO................3 | \|___\| |
